# A Genotype-Phenotype Correlation Study of Exon Skip-Equivalent In-Frame Deletions and Exon Skip-Amenable Out-of-Frame Deletions across the *DMD* Gene to Simulate the Effects of Exon-Skipping Therapies: A Meta-Analysis

**DOI:** 10.3390/jpm11010046

**Published:** 2021-01-14

**Authors:** Saeed Anwar, Merry He, Kenji Rowel Q. Lim, Rika Maruyama, Toshifumi Yokota

**Affiliations:** 1 Department of Medical Genetics, Faculty of Medicine and Dentistry, University of Alberta, Edmonton, AB T6G 2H7, Canada; sanwar@ualberta.ca (S.A.); merry1@ualberta.ca (M.H.); kenjirow@ualberta.ca (K.R.Q.L.); yokotama@ualberta.ca (R.M.); 2 The Friends of Garrett Cumming Research and Muscular Dystrophy Canada, HM Toupin Neurological Science Research Chair, Edmonton, AB T6G 2H7, Canada

**Keywords:** dystrophinopathy, duchenne muscular dystrophy (DMD), becker muscular dystrophy (BMD), dystrophin, reading frame rule, exon skipping, skip-equivalent deletions

## Abstract

Dystrophinopathies are caused by mutations in the *DMD* gene. Out-of-frame deletions represent most mutational events in severe Duchenne muscular dystrophy (DMD), while in-frame deletions typically lead to milder Becker muscular dystrophy (BMD). Antisense oligonucleotide-mediated exon skipping converts an out-of-frame transcript to an in-frame one, inducing a truncated but partially functional dystrophin protein. The reading frame rule, however, has many exceptions. We thus sought to simulate clinical outcomes of exon-skipping therapies for *DMD* exons from clinical data of exon skip-equivalent in-frame deletions, in which the expressed quasi-dystrophins are comparable to those resulting from exon-skipping therapies. We identified a total of 1298 unique patients with exon skip-equivalent mutations in patient registries and the existing literature. We classified them into skip-equivalent deletions of each exon and statistically compared the ratio of DMD/BMD and asymptomatic individuals across the *DMD* gene. Our analysis identified that five exons are associated with significantly milder phenotypes than all other exons when corresponding exon skip-equivalent in-frame deletion mutations occur. Most exon skip-equivalent in-frame deletions were associated with a significantly milder phenotype compared to corresponding exon skip-amenable out-of-frame mutations. This study indicates the importance of genotype-phenotype correlation studies in the rational design of exon-skipping therapies.

## 1. Introduction

Dystrophinopathies are a spectrum of X-linked muscular dystrophies caused by mutations in the *DMD* gene encoding the dystrophin protein, which helps maintain the integrity of muscle membranes [1]. The most lethal end of this spectrum, Duchenne muscular dystrophy (DMD), generally arises from mutations that disrupt the translational reading frame and result in an absence of dystrophin [2]. Three other major conditions that belong to this spectrum are Becker muscular dystrophy (BMD, a mild form of DMD), intermediate muscular dystrophy (IMD, an intermediate form between DMD and BMD), and DMD-associated dilated cardiomyopathy (DCM, a type of nonischemic heart-disease) [2].

With 79 constitutive exons and (at least) seven alternatively-used exons, the *DMD* gene is one of the largest known genes in the human genome [2,3]. Because of the enormous length of the gene, it is highly vulnerable to mutations and, one out of three mutations are de novo in nature. Besides, the presence of two mutational hotspots, encompassing exons 3–22 and exons 45–55, within the coding sequence of the gene makes it more vulnerable to mutations [4,5,6]. To date, over five thousand *DMD* mutations have been reported. Large deletions involving deletion of one or more exons account for most cases (~68%), while duplications, other small mutations, and rarely deep intronic copy number variations cause the rest of the cases [7,8,9]. The major determinant of phenotypes is the reading frame rule, i.e., the mutations giving rise to an mRNA with disrupted reading frame (out-of-frame) result in a severe pathology, i.e., DMD. On the other hand, in-frame deletions result in the production of truncated yet (partially) functional dystrophins, causing a mild clinical phenotype called BMD [10]. The reading frame rule is accurately predictive in ~90% of DMD cases; however, not as consistent for BMD cases, with prediction rates ranging from 56–91% in different cohorts [8,11].

This reading frame rule provides the rationale for a therapeutic strategy, called therapeutic exon skipping using antisense oligonucleotides to transform DMD-related out-of-frame mRNAs into in-frame ones to produce truncated dystrophin, against severe and lethal cases of DMD. Recently, three exon-skipping oligonucleotide drugs, namely eteplirsen, golodirsen, and viltolarsen, received accelerated approval by the U.S. Food and Drug Administration (FDA), and a few other oligonucleotide-based drugs are in later stages of clinical trials [12,13,14]. As such, antisense oligonucleotide-mediated single exon skipping has great promise for treating DMD effectively. However, because of these drugs’ highly specific nature, they apply to only a small portion of the DMD population, e.g., eteplirsen, golodirsen, and viltolarsen together are applicable for a total of around 20% of the entire DMD population in the U.S. [12,13]. Recently, skipping of multiple exons, e.g., exons 45–55, using mutation-tailored cocktails of antisense oligonucleotides have shown potential for clinical application, and the applicability of such cocktail drug was shown to reach over 65% of DMD patients with large deletions [6,15].

The reading frame rule, however, is not always an accurate predictor of clinical phenotypes [16]. As multiple exonic deletions are amenable to skipping of each exon, the quasi-dystrophins resulting from the various exon skipped transcripts may vary in stability, function, and phenotype. The DMD/BMD phenotypic ratio observed in individuals with confirmed in-frame deletions starting and/or ending at frameshifting exons have been examined to estimate the therapeutic outcomes from skipping several exons [17,18,19]. In this study, we employed meta-analysis on the published literature and databases containing data on the genotype-phenotype association. It provides an overview of clinical presentations in patients with each exon ”skip-equivalent” deletion and determines the best estimate of each exon skip-amenable mutation’s clinical outcome of exon-skipping therapies.

## 2. Materials and Methods

### 2.1. Clinical Database Search and Inclusion/Exclusion Criteria

The French UMD-DMD-France database and the eDystrophin are two online registries for individuals and families affected by dystrophinopathies [20,21]. From 30 March 2020 to 30 July 2020, we queried these two databases for relevant cases. We excluded patients with pending clinical phenotypes and female carriers from the analysis. If a record were present in both databases, the data were entered only once into the data tabulation sheet.

### 2.2. Literature Search and Inclusion/Exclusion Criteria

To collect more data on the clinical features of genetically confirmed DMD patients, a literature search of PubMed was conducted using the following query terms: Dystrophin*[Title/Abstract]) OR (Duchenne[Title/Abstract])) OR (Becker*[Title/Abstract])) OR (muscular dystrophy[Title/Abstract]) OR (mutation[Title/Abstract])) OR (large deletion[Title/Abstract])) OR (mutation spectrum[Title/Abstract])) OR (MLPA[Title/Abstract])) OR (ligation[Title/Abstract])) OR (CGH[Title/Abstract])) OR (comparative genomic hybridization[Title/Abstract])). We then also searched google scholar using an equivalent query.

Two researchers (S.A. and M.H.) independently reviewed titles and abstracts of the identified articles to determine that only relevant publications were included. Articles were excluded if, after review, it was evident that the article discussed a non-human disease model, diseases other than dystrophinopathies, a non-dystrophin study, or presents a meta-analysis. We excluded the manuscripts presenting inadequate genotype-phenotype information or discussing only female carriers. Additionally, we excluded the studies that were included in any of the two databases mentioned above. In addition, only the manuscripts reporting cases of dystrophinopathies detected by multiplex ligation-dependent probe amplification (MLPA), comparative genomic hybridization (CGH), or equivalent diagnostic procedures were included. No restrictions were made based on language, publication year, publication status, and the latest search date.

**Figure 1:**
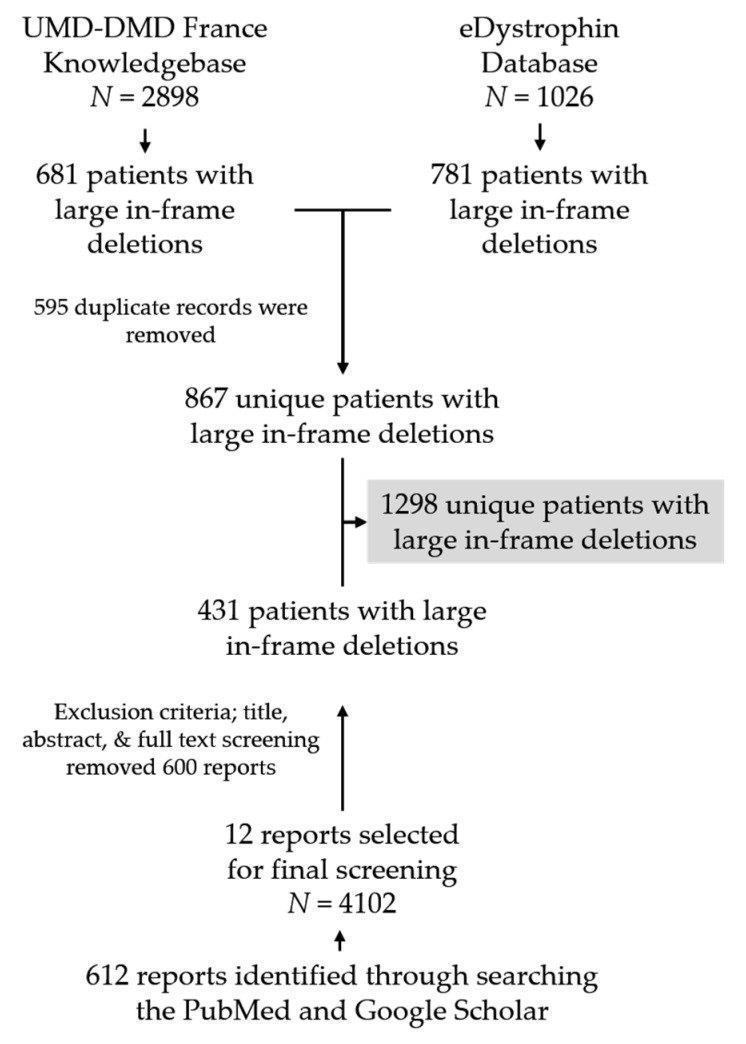
Flow chart showing database screening and literature search procedure to collect patients’ clinical information with confirmed DMD large deletions. *N* indicates the number of individuals present in each data source.

We identified a total of 612 unique articles by searching PubMed and Google Scholar, out of which 12 articles were finally selected based on our exclusion criteria after the title and abstract review. We then conducted a comprehensive review of these 12 articles to identify individuals with genetically confirmed large deletions in *dystrophin* (Figure 1, Figure S1, Table S1).

### 2.3. Data Extraction and Analysis of the Data

Two authors (S.A. and M.H.) independently reviewed the full-text versions and, if available, the Supplementary Materials to extract clinical data of dystrophinopathy patients with in-frame deletions from the literature. Data of patients with deletions involving exons 1 and 79 were not included as deletions of these two exons would not produce truncated dystrophins. To assess duplicate records in multiple databases and literature, we utilized the following strategy. One of the investigators (S.A.) compared the partially de-identified information (identifiers, country origin, age at diagnosis, date of birth, and available mutation information, e.g., mutation start point and end point, and type of mutation), assuming any records with these same items represented the same patient [22,23]. Another author (K.R.Q.L.) scrutinized the inclusion of mutation information and the removal of duplicate records. We categorized the clinically confirmed patients into two groups: (i) DMD, and (ii) BMD and asymptomatic. We categorized BMD and asymptomatic cases together because exon-skipping therapies aim to convert lethal DMD cases into milder forms, whether BMD or asymptomatic, with in-frame deletions. In addition, those asymptomatic cases at the time of examination could become BMD at a later age. The asymptomatic cases, therefore, are combined with BMD in a single group.

### 2.4. Exon Skip-Amenable and Exon Skip-Equivalent Mutations

We defined exon skip-amenable mutation as follows; (1) for frame-shifting exons (i.e., exons 2, 6, 7, 8, 11, 12, 17, 18, 19, 20, 21, 22, 43, 44, 45, 46, 50, 51, 52, 53, 54, 55, 56, 57, 58, 59, 61, 62, 63, 65, 66, 67, 68, 69, 70, 75, 76, and 78), we included large out-of-frame deletion mutations that can be made in-frame by skipping of exon X as “exon X skip-amenable mutations”. For example, exon 52 of DMD is a frameshifting exon, and skipping of an adjacent exon, exon 51 or exon 53, theoretically restores the reading frame (exon 51 skip-amenable and exon 53 skip-amenable), (2) for non-frame-shifting exons (all other exons between exons 2–78), we included nonsense mutations and small out-of-frame indels in exon Y as ”exon Y skip-amenable mutations”. For example, therapeutic skipping of exon 23 for a nonsense mutation in exon 23 would restore the reading frame, leading to truncated dystrophin protein expression (exon 23 skip-amenable). We included patients who naturally harbor large in-frame deletion mutations starting and/or ending at exon Z as “exon Z skip-equivalent mutation”. Deletions starting at exon 1 and ending at exon 79 are not included.

The same overall definitions of exon skip-amenable and exon skip-equivalent mutations can be applied for multi-exon regions, e.g., exons 3–9 or 45–55. Mutations (i.e., large deletions, large duplications, small indels, and nonsense) whose disrupted reading frame can be restored by multi-exon skipping are considered multi-exon skip-amenable. Naturally, mutations involving both frame-shifting or non-frame-shifting exons are included in our counts. Mutations partly outside of exons 3–9 and 45–55 are not included in exons 3–9 and 45–55 skip-amenable mutations, respectively (e.g., del. ex 43–45, del. ex 51–57). On the other hand, naturally occurring mutations (e.g., in-frame large deletions of exons 3 to 9 and exons 45 to 55) that model multi-exon-skipped transcripts are considered multi-exon skip-equivalent.

### 2.5. Statistical Analysis

The odds of DMD for a given exon were compared to BMD and asymptomatic phenotype’s odds using Fisher’s exact test. The ratios of DMD to BMD/asymptomatic phenotypes associated with a specific mutation and all other mutations were compared using Fisher’s exact test. The test-statistic values of multiple test comparisons were adjusted using the Benjamini–Hochberg method to control false discovery rates [24]. To identify if there were hotspot regions in the gene, we compared the frequency of in-frame deletions starting and/or ending at a given exonic region and all other exons using f-statistic. Additionally, we compared the phenotypic outcomes associated with out-of-frame mutations amenable to exon skipping (exon skip-amenable) and exon skip-equivalent in-frame deletions of each exon using Fisher’s exact test. Since the databases included and the manuscripts reviewed were, in general, descriptive, case-specific or focus group studies rather than randomized ones, we could not do a formal risk of bias assessment on the data. All data were analyzed in Microsoft Excel (Office 365, 2019).

## 3. Results

### 3.1. Patient Pool and Deletional Patterns

The UMD-DMD France and eDystrophin databases contain clinical data of 681 and 781 individuals, respectively, with confirmed clinical outcomes with in-frame exon deletions within *dystrophin*. Data of 595 individuals (29 DMD, 556 BMD, and 10 asymptomatic individuals) was included in both databases. In addition, our literature search identified clinical data of 431 unique patients with in-frame deletions. The final patient pool we analyzed for clinical information contained information of 1298 individuals with confirmed clinical outcomes resulting from genetically diagnosed dystrophinopathy, including 277 (21.34%) individuals with DMD and 1021 (78.66%) with milder BMD (*n* = 976) or asymptomatic phenotypes (*n* = 45) (Figure S2A). There was a significant difference in the ratio of DMD to BMD and asymptomatic phenotypes between the records obtained from the two databases and literature searching (eDystrophin vs. literature search: *p*-value < 0.0001, 95% CI = 27.60–37.79; UMD-DMD France database vs. literature search: *p*-value < 0.001, 95% CI = 30.49–40.55; UMD-DMD France database vs. eDystrophin: *p*-value = 0.0519, 95% CI = −0.034–5.65) (Figure S2A).

Theoretically, there are 1408 potential large in-frame deletions possible across the *DMD* gene (Table S2). Our patient pool represented 180 (12.78%) of these potential in-frame deletions (Figure S2B). Of these theoretically possible large in-frame deletions, Δ 45–47 (*n* = 317, 23 DMD) and Δ 45–48 (*n* = 205, 19 DMD) were the most common deletional patterns reported among the patients. Two deletional hotspots were identified between exons 3 and 13, and 44 and 55, as 15.58% and 74.46% deletions started and/or ended within these two regions (*p*-value < 0.0001, for both regions) (Figures S2C and S3).

### 3.2. More BMD and Asymptomatic Phenotypes Were Associated with In-Frame Deletions

**Figure 2:**
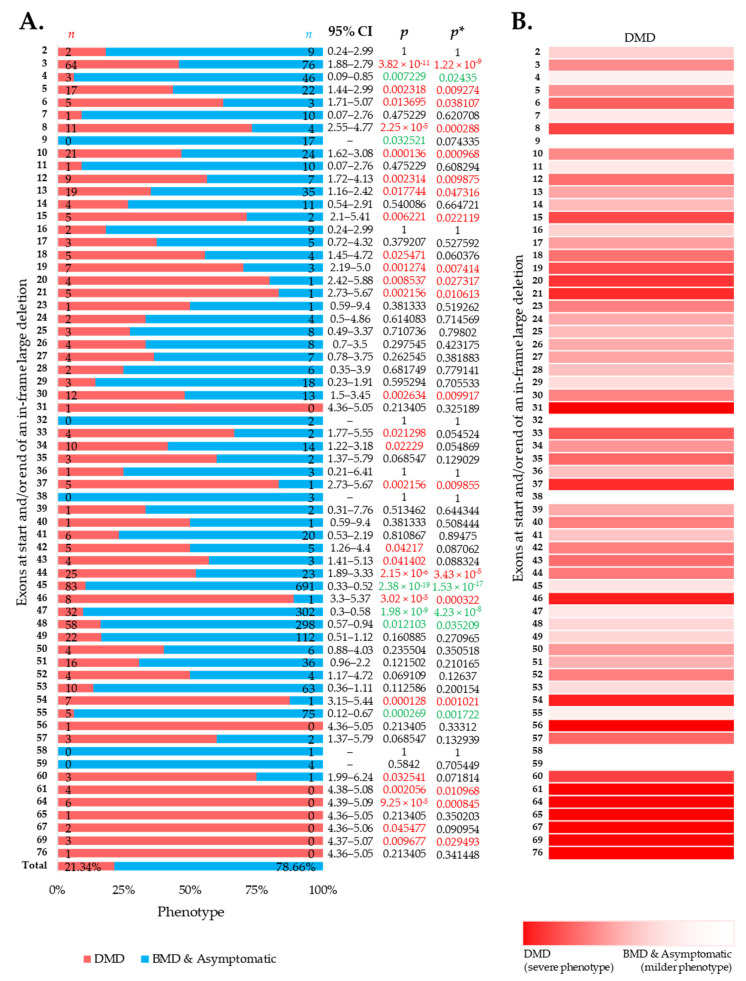
Clinical phenotypes of exon skip-equivalent in-frame *DMD* exon deletions. (**A**) Association between in-frame deletions starting and/or ending at each exon (exon skip-equivalent) and consequent phenotypes. Phenotypic ratios associated with in-frame deletions starting and/or ending at a given exon and all other exons were compared using Fisher’s exact test. *n* indicates the number of individuals with DMD (red) and milder (blue; BMD and asymptomatic) phenotypes. Green and red color indicate a significantly lower and higher incidence of DMD phenotype for a given exon, respectively, as compared to the overall incidence rate. *p* = *p*-value, as calculated by Fisher’s exact test; p* = Benjamini–Hochberg adjusted *p*-value. (**B**) Heatmap showing the relative severity of the consequence of in-frame deletions starting and/or ending at specific exons.

Large in-frame deletions were associated with 9.73%, 6.90%, and 42.46% cases with a severe DMD phenotype in the eDystrophin, UMD-DMD France databases, and the literature (Figures S4–S6). Overall, the reading frame rule was predictive for nearly 78.54% of the cases with large in-frame deletions (Figure 2A). However, exclusive of the literature’s records, the prediction rate was 88.95% (Figures S4–S6). We identified 19 exons in-frame deletions, resulting in a significantly severer phenotype than average (Figure 2A,B). Additionally, in-frame deletions starting and/or ending at five exons, including exon 4, 45, 47, 48, and 55, are deemed to result in milder phenotypes (Figure 2A). In addition, we observed a lower incidence of DMD phenotype associated with in-frame deletions starting and/or ending at exons encoding the dystrophin protein’s central rod domain (Figure S7). On the other hand, in-frame deletions starting and/or ending at the extreme ends of the protein was associated with more DMD phenotype (Figure S7).

### 3.3. Distribution of Phenotypes for Exon Skip-Amenable Mutations to Each Exon

**Figure 3:**
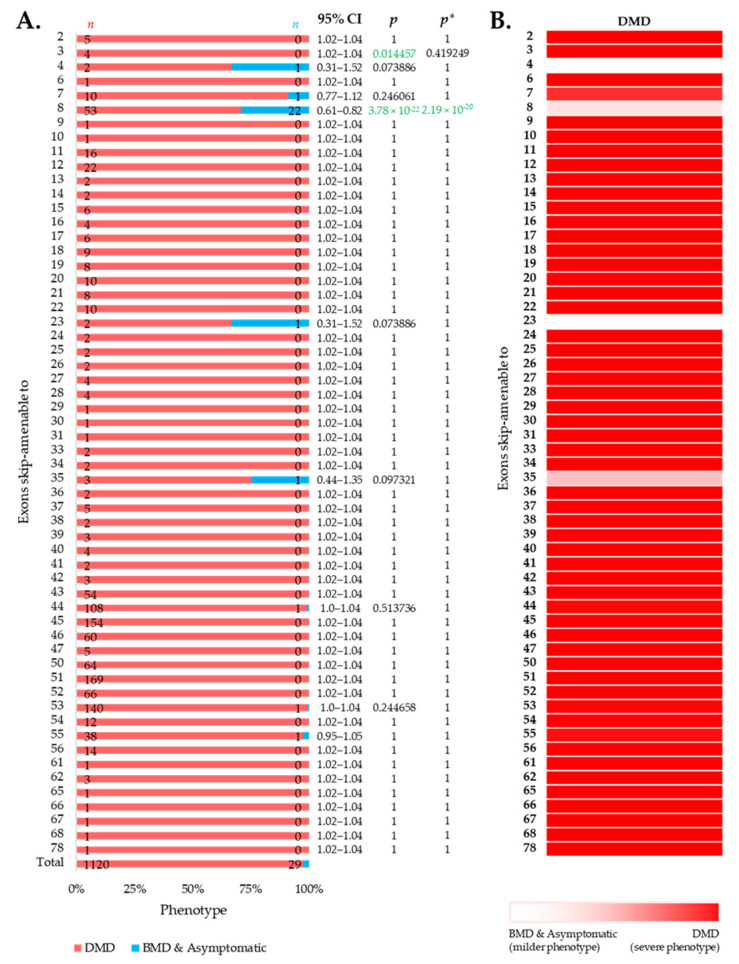
Phenotypic outcomes of exon skip-amenable mutations present in the UMD-DMD France database. (**A**) Distribution of phenotypes for each exon skip-amenable mutation. Phenotypic ratios associated with exon skip-amenable mutations for each exon vs. all other exons are compared using Fisher’s exact test. *n* indicates the number of individuals with DMD (red) and milder (blue; BMD and asymptomatic) phenotypes. Green color indicates a significantly lower incidence of DMD phenotype. *p* = *p*-value, as calculated by Fisher’s exact test; p* = Benjamini–Hochberg adjusted *p*-value. (**B**) Heatmap showing the relative severity of the consequence of out-of-frame mutations amenable to skipping of each exon.

We then intended to look at the clinical phenotypes associated with out-of-frame mutations that can be converted to in-frame by therapeutic skipping of one (or more) exons, i.e., skip-amenable mutations, using the patient data collected from the UMD-DMD France Knowledgebase. We determined the ratio of individuals with severe and milder phenotypes associated with exon skip-amenable mutations. Collectively, our analysis identified a total of 1149 individuals with single exon skip-amenable mutations, including 1120 (97.476%) patients with a severe phenotype, i.e., DMD (Figure 3).

### 3.4. Comparison of Phenotypes of In-Frame Exon Skip-Equivalent and Out-of-Frame Exon Skip-Amenable Mutations

**Figure 4:**
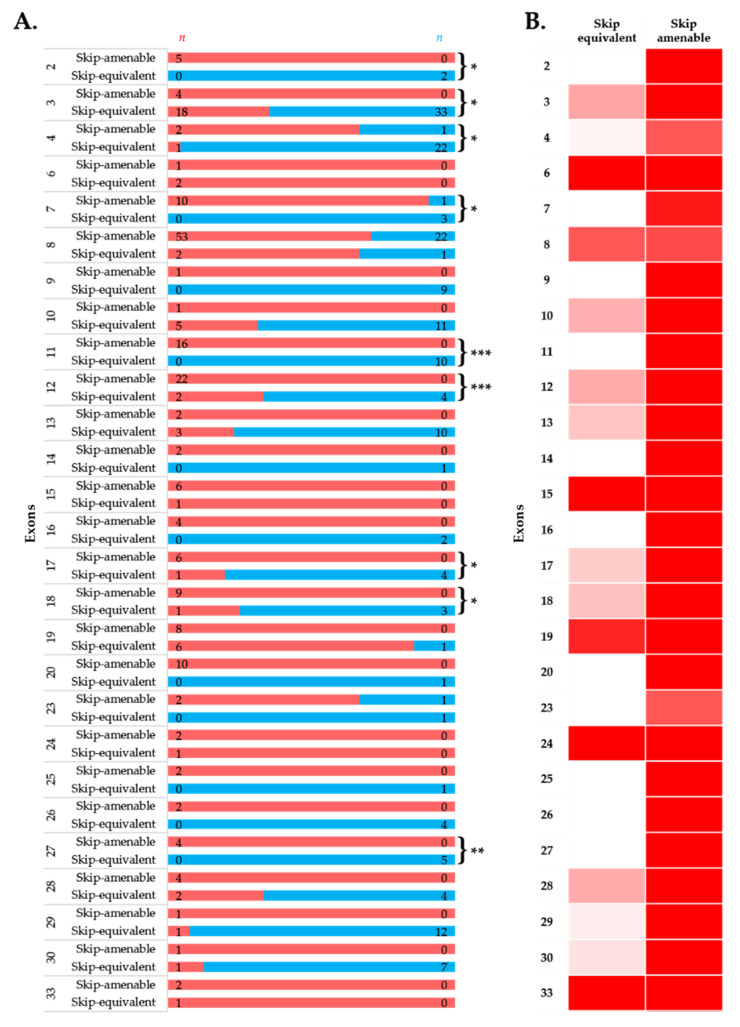

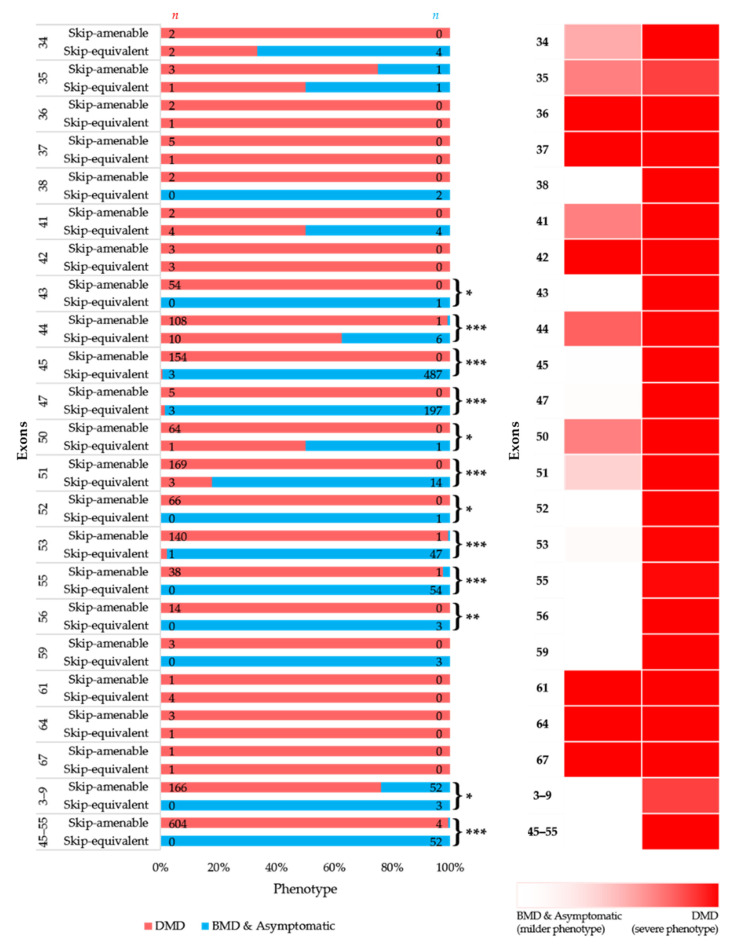
Comparison of clinical phenotypes associated with out-of-frame mutations amenable to exon skipping in UMD-DMD database and in-frame exon skip-equivalent deletions of each exon to simulate the effects of exon-skipping therapies. (**A**) Phenotypic outcomes associated with mutations amenable to exon skipping and exon skip-equivalent in-frame deletions of each exon and their consequent phenotypes. *n* indicates the number of individuals with DMD (red) and milder (blue; BMD and asymptomatic) phenotypes. Asterisks indicate that exon skip-equivalent in-frame deletions are associated with a significantly milder phenotype compared to corresponding exon skip-amenable out-of-frame mutations. We compared the incidence of DMD associated with exon skip-equivalent (or group of exons, e.g., exons 3–9, and exons 45–55) and mutations amenable to skipping each exon (or group of exons, e.g., exons 3–9, and exons 45–55). The statistical significance was calculated using Fisher’s exact test. (**B**) Heatmap showing the relative severity of the consequence of exon skip-equivalent in-frame deletions and exon skip-amenable mutations. * *p* < 0.05, ** *p* < 0.01, *** *p* < 0.001

To simulate the clinical phenotype before and after exon-skipping therapy, we looked at the differences in phenotypic outcomes of in-frame exon skip-equivalent and out-of-frame exon skip-amenable mutations for each exon using the data present in the UMD-DMD France Knowledgebase. Our analysis identified 21 exons at which in-frame deletions start and/or end (exon skip-equivalent mutations) were associated with a significantly lower DMD incidence compared to corresponding exon skip-amenable mutations (Figure 4A,B). Additionally, exons 3–9 and exons 45–55 skip equivalent deletions were associated with a significantly lower incidence of a severe phenotype compared to exons 3–9 and exons 45–55 skip-amenable mutations (*p*-value < 0.0001).

## 4. Discussion

The FDA approvals of eteplirsen, viltolarsen, and golodirsen are an inspiring development in treating DMD, although these drugs cumulatively can only be administered to approximately 20% of the total DMD population who are specifically amenable to exon 51 and exon 53 skipping [12,25,26]. Besides, the development of antisense therapies to skip additional exons could theoretically help to treat up to ~80% of individuals living with DMD [16,27]. Currently, clinical trials to evaluate exon 44, 45, 51, and 53 skipping antisense drugs are underway (clinicaltrials.gov: NCT02530905, NCT04179409, NCT02667483, NCT02500381, NCT04433234, NCT04129294). Besides, the development of mutation-tailored cocktail antisense drugs applicable for treating a large portion of the DMD population carrying out-of-frame deletions also showed potential [15,28].

This study focused on the phenotypes associated with large deletions involving one or more exons to result in in-frame transcripts. While 1408 large in-frame deletion patterns are theoretically possible within the *DMD* gene, this study identified only 180 (12.78%) of them in individuals in the searched sources of clinical data (Figure S2B and Table S1). The absence of clinical data on individuals with the remaining deletions, perhaps due to the deletions’ extreme rarity or that the associated phenotypes are asymptomatic or very mild, that the mutations remain undetected and unreported.

The clinical data on individuals with large in-frame deletions in *DMD* present that nearly 80% (78.54%) have milder BMD or asymptomatic phenotypes (Figure S2A and Figure 2A). Nearly 1 out of 5 individuals (21.46%) developed severe phenotype, i.e., DMD, despite their predicted in-frame deletions. Interestingly, the occurrence of severe phenotype was over four-times higher among the individuals constituted from the literature (Figure S2A). This indicates the potential presence of reporting bias in the clinical reports [29]. To note, in the aggregate of the data collected from the two databases but without those obtained from the literature, 11.05% of individuals with in-frame large deletions developed severe phenotype, which is comparable with the numbers indicated by previous reports (Figures S5 and S6) [7,8,10,20,30].

A previous report studying the clinical phenotypes of *DMD* exon 51 skip-equivalent deletions identified 12% of patients had severe phenotypes despite their predicted in-frame deletions [31]. The individuals with DMD phenotype with in-frame deletions indicate the need for subtle knowledge of the reading-frame rule for dystrophinopathies. It also reflects the complex biology of dystrophinopathies and an array of different factors may influence the ultimate clinical phenotype. These factors may include but are not limited to the inherent leakiness of splicing seen in some exons, the effect of predicted frame-altering mutations on splicing signals, and the stability and tertiary structure of the resultant protein [16,32,33,34].

Irrespective of the data source, deletions starting and/or ending at some exons were associated with a more severe phenotype (Figure 2 and Figures S2–S6). Additionally, in-frame deletions starting and/or ending within the exons 3–13 hotspot region was associated with an elevated frequency of a severe phenotype, while those starting and/or ending within exons 44–55 region resulted in a significantly lower incidence rate for a DMD phenotype (Figure S2C and Figure 2). An explanation for why the occurrence of a severe phenotype is associated with these deletions is currently unclear. One possible reasoning could be that these deletions, although been predicted as in-frame, result in a less stable or non-functional protein, as a part of N-terminal hotspot encodes for the actin-binding domain. However, the deletion of certain exons involved with coding for portions of the central rod domain may have a less significant impact on skeletal muscle pathology [35], which is what is reflected in this study. Given the retrospective and meta-analytic nature of this study, we did not have access to do any muscle tissue biopsy to investigate the actual cause beneath the elevated frequency of severe phenotype associated with these deletions, and it would also be beyond the scope of this study. Further assessment in clinical status and muscle tissue analysis may help conclude the factors behind the findings of a severe phenotype associated with these exons, which could be interesting and may provide essential insights for therapeutic development.

Importantly, this study identified five exons, including exons 4, 45, 47, 48, and 55, at the start and/or end of in-frame deletions associated with a significantly lower frequency of a DMD phenotype than average (Figure 2A). A previous study of 4894 patients with in-frame and out-of-frame large deletions from the TREAT-NMD DMD Global database listed the top ten skippable *DMD* exons, including exons 8, 43, 44, 45, 50, 51, 52, 53, and 55, that would apply to the largest group of patients with DMD [7]. Our study identified that individuals with large in-frame deletions starting and/or ending at many of these exons had a significantly lower frequency of developing a severe phenotype than those having a mutation amenable to skipping those exons (Figure 2 and Figure 4). It also suggests that the skipping of exons 3 to 9 and 45 to 55 could be promising targets for treating DMD (*p*-value < 0.0001).

Among the exons we identified as associated with a significantly lower incidence of DMD, exon 45 is already being thoroughly studied as a target for the development of exon-skipping therapies [16,36]. Casimersen, an antisense drug of phosphorodiamidate morpholino chemistry to treat DMD amenable to exon 45 skipping, has recently been accepted and placed under priority review by the FDA [37,38]. The other five exons are also deemed promising therapeutic targets of exon skipping, although some of them are not applicable to many patients.

While the present study included a reasonably comprehensive dataset, it has several limitations. First, a significant limitation is that the specific deletional patterns between DMD patients eligible for exon skipping with out-of-frame deletions and in-frame deletions of exon skip-equivalent deletion mutations are not always comparable. This is particularly the case for exon 44 skipping. Over two-thirds of the (67.89%) out-of-frame deletions amenable to exon 44 skipping start at exon 45, whereas ~98% of the in-frame deletions (exon 44 skip-equivalent) end at exon 44 (Figure S8). Second, the effects of in-frame deletions at the DNA level are not necessarily equivalent to the effects at the RNA level in antisense-treated cells. For example, in a DMD dog model, skipping of exon 8 using an antisense morpholino led to spontaneous skipping of exon 9 in addition to exon 8 [28]. As such, the effects of exon 8 skipping might be more relevant to exon 9 skip-equivalent in-frame deletion rather than exon 8 skip-equivalent in-frame deletion. Third, among the literature included, some studies only enrolled patients diagnosed with either DMD or BMD, potentially influencing the results. Fourth, some studies did not interrogate all *DMD* exons, leaving the possibility that, although unlikely, the patients may have harbored a second mutation. Fifth, the exceedingly high proportion of the DMD phenotype in the literature also raises the concern that there could be some reporting biases in play. Lastly, other factors, including the variability of antisense oligonucleotide-mediated exon skipping efficacy for different exons, also need to be considered to design exon-skipping therapy. Hence, these potential limitations should be considered with care when interpreting the data presented in this study.

## 5. Conclusions

This study highlights the genotype-phenotype association among individuals with large in-frame deletions starting and/or ending at different exons in *DMD*. While most exon skip-equivalent in-frame deletions are associated with a significantly milder phenotype compared to corresponding exon skip-amenable out-of-frame mutations, we identified several exon skip-equivalent in-frame deletions that are the most promising therapeutic targets. However, the phenotypic variability in individuals with specific in-frame exon deletions found in this study is suggestive of the issue that the response to exon-skipping therapy could be variable and may be impacted by multiple factors. This study largely indicates that genotype-phenotype correlation analysis can significantly contribute to the rational design of exon-skipping therapies; however, hints at the necessity of continued evaluation of genetic and other modifiers.

## Data Availability

The data prepared for this study are available in the article or Supplementary Materials.

## Supplementary Materials

The following are available online at https://www.mdpi.com/2075-4426/11/1/46/s1, Figure S1: Deletional pattern in the DMD gene. From left to right on the bottom row represents the number of exons (2–78) in the DMD gene. The black shaded area shows the range of each deletion in the *DMD* gene. Figure S2: Patient pool and dystrophin mutational patterns among the individuals with large in-frame deletions. Figure S3: Frequency of deletions starting and/or ending at a given exon was significantly high in a proximal (exons 3 to 13, *p*-value = 0.000015, f = 20.64776) and a distal (exons 44 to 55, *p*-value = 0.000069, *f* = 17.2277) region. Figure S4: Association between in-frame deletions (A) starting, (B) ending, and (C) starting and/or ending at each exon and consequent phenotypes in the eDystrophin database. Figure S5: Association between in-frame deletions (A) starting, (B) ending, and (C) starting and/or ending at each exon and consequent phenotypes in the UMD-DMD France database. Figure S6: Association between in-frame deletions (A) starting, (B) ending, and (C) starting and/or ending at each exon and consequent phenotypes in the records identified from existing literature; Figure S7: Overview of clinical phenotypes associated with in-frame deletions starting and/or ending at different exons and their corresponding dystrophin domains. Figure S8: Phenotypes associated with different exon 44 skip equivalent and amenable to exon 44 skipping mutations. Table S1: Clinical data obtained from literature searching. Table S2: List of all theoretically possible in-frame deletion within *dystrophin*.
