# Supplementary material for: A Genotype-Phenotype Correlation Study of Exon Skip-Equivalent In-Frame Deletions and Exon Skip-Amenable Out-of-Frame Deletions across the DMD Gene to Simulate the Effects of Exon-Skipping Therapies: A Meta-Analysis
Source: J Pers Med. 2021 Jan 14;11(1):46. doi: 10.3390/jpm11010046 (PMC7830903; doi:10.3390/jpm11010046)
Supplement: Supplementary file 1 [file jpm-11-00046-s001.zip › Figure S1.pdf]

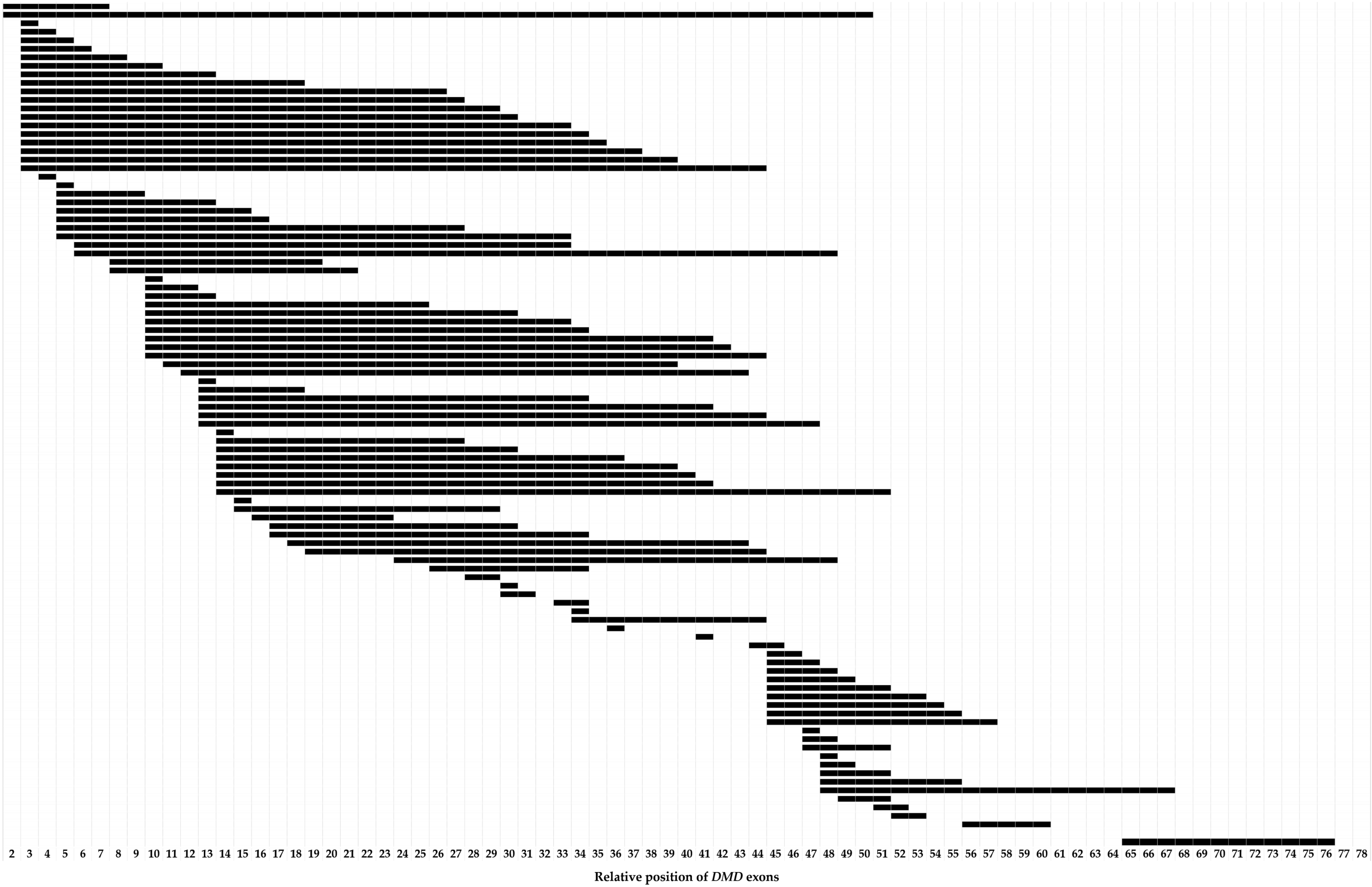

Figure S1: Deletional pattern in the *DMD* gene. From left to right on the bottom row represents the number of exons (2–78) in the *DMD* gene. The black shaded area shows the range of each deletion in *DMD* gene.
