# Supplementary material for: A Genotype-Phenotype Correlation Study of Exon Skip-Equivalent In-Frame Deletions and Exon Skip-Amenable Out-of-Frame Deletions across the DMD Gene to Simulate the Effects of Exon-Skipping Therapies: A Meta-Analysis
Source: J Pers Med. 2021 Jan 14;11(1):46. doi: 10.3390/jpm11010046 (PMC7830903; doi:10.3390/jpm11010046)
Supplement: Supplementary file 1 [file jpm-11-00046-s001.zip › Figure S2.pdf]

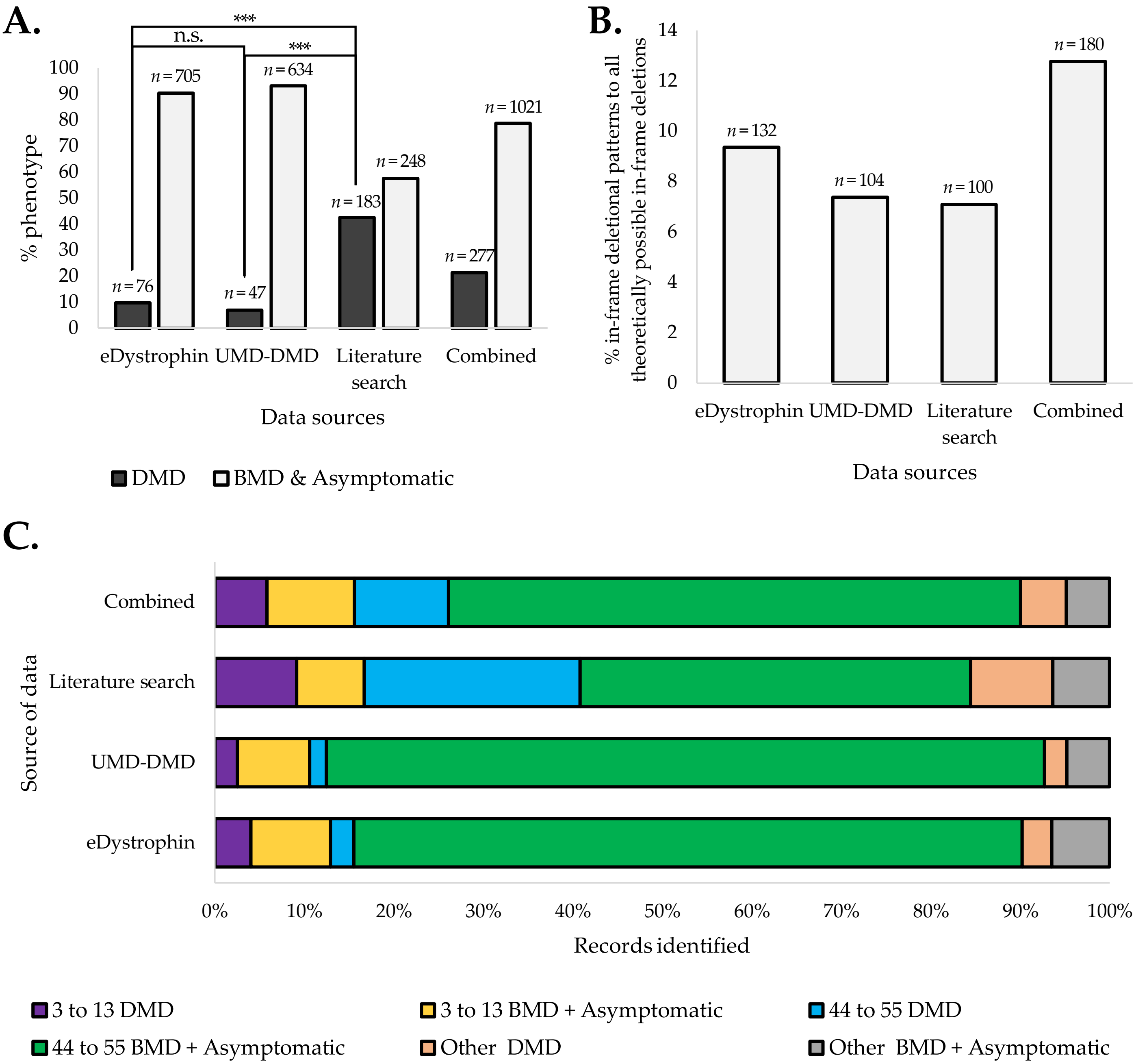

Figure S2. Patient pool and *dystrophin* mutational patterns among the individuals with in-frame large deletions. (**A**) Sources of the clinical data on individuals with in-frame large deletions in *dystrophin*. (**B**) The proportion of deletions present to all theoretically possible in-frame large deletions ($N = 1408$) in each data source. (**C**) Frequency of in-frame large deletions starting or ending at exons 3 to 13 and exons 44 to 55 regions, compared to all other exons. $^{***}p < 0.0001$, n.s. indicates a non-significant difference.
