# Supplementary material for: A Genotype-Phenotype Correlation Study of Exon Skip-Equivalent In-Frame Deletions and Exon Skip-Amenable Out-of-Frame Deletions across the DMD Gene to Simulate the Effects of Exon-Skipping Therapies: A Meta-Analysis
Source: J Pers Med. 2021 Jan 14;11(1):46. doi: 10.3390/jpm11010046 (PMC7830903; doi:10.3390/jpm11010046)
Supplement: Supplementary file 1 [file jpm-11-00046-s001.zip › Figure S3.pdf]

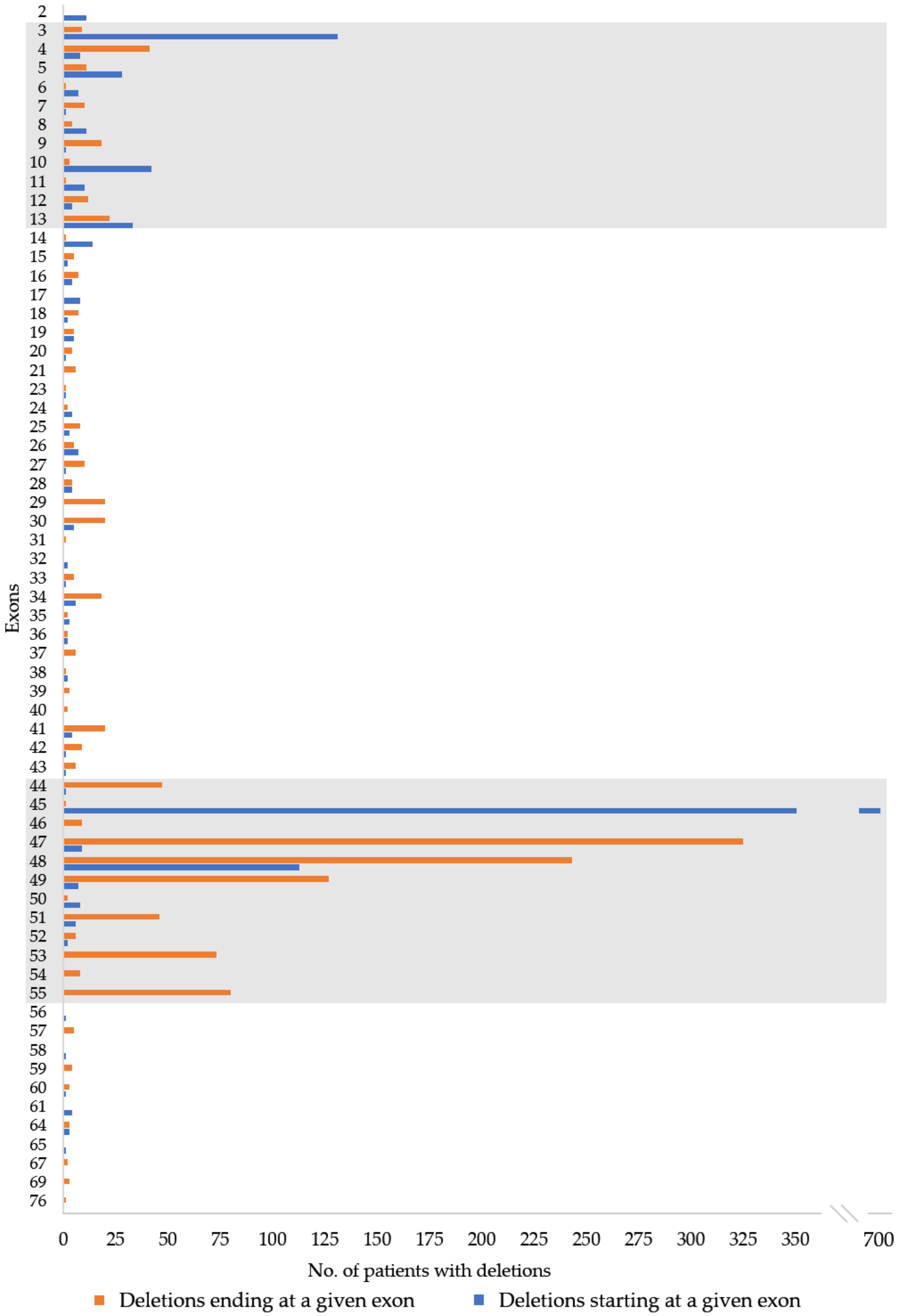

Figure S3. Frequency of deletions starting and/or ending at a given exon was significantly high in a proximal (exons 3 to 13, p-value = 0. 000015, f = 20.64776) and a distal (exons 44 to 55, p-value = 0.000069, f = 17.2277) region. 88.12% of individuals harbored deletions starting and/or ending within these two regions of the dystrophin. The average frequency of in-frame deletions starting and/or ending at a given region and all other exons was compared using f-statistic.
