# Supplementary material for: A Genotype-Phenotype Correlation Study of Exon Skip-Equivalent In-Frame Deletions and Exon Skip-Amenable Out-of-Frame Deletions across the DMD Gene to Simulate the Effects of Exon-Skipping Therapies: A Meta-Analysis
Source: J Pers Med. 2021 Jan 14;11(1):46. doi: 10.3390/jpm11010046 (PMC7830903; doi:10.3390/jpm11010046)
Supplement: Supplementary file 1 [file jpm-11-00046-s001.zip › Figure S4.pdf]

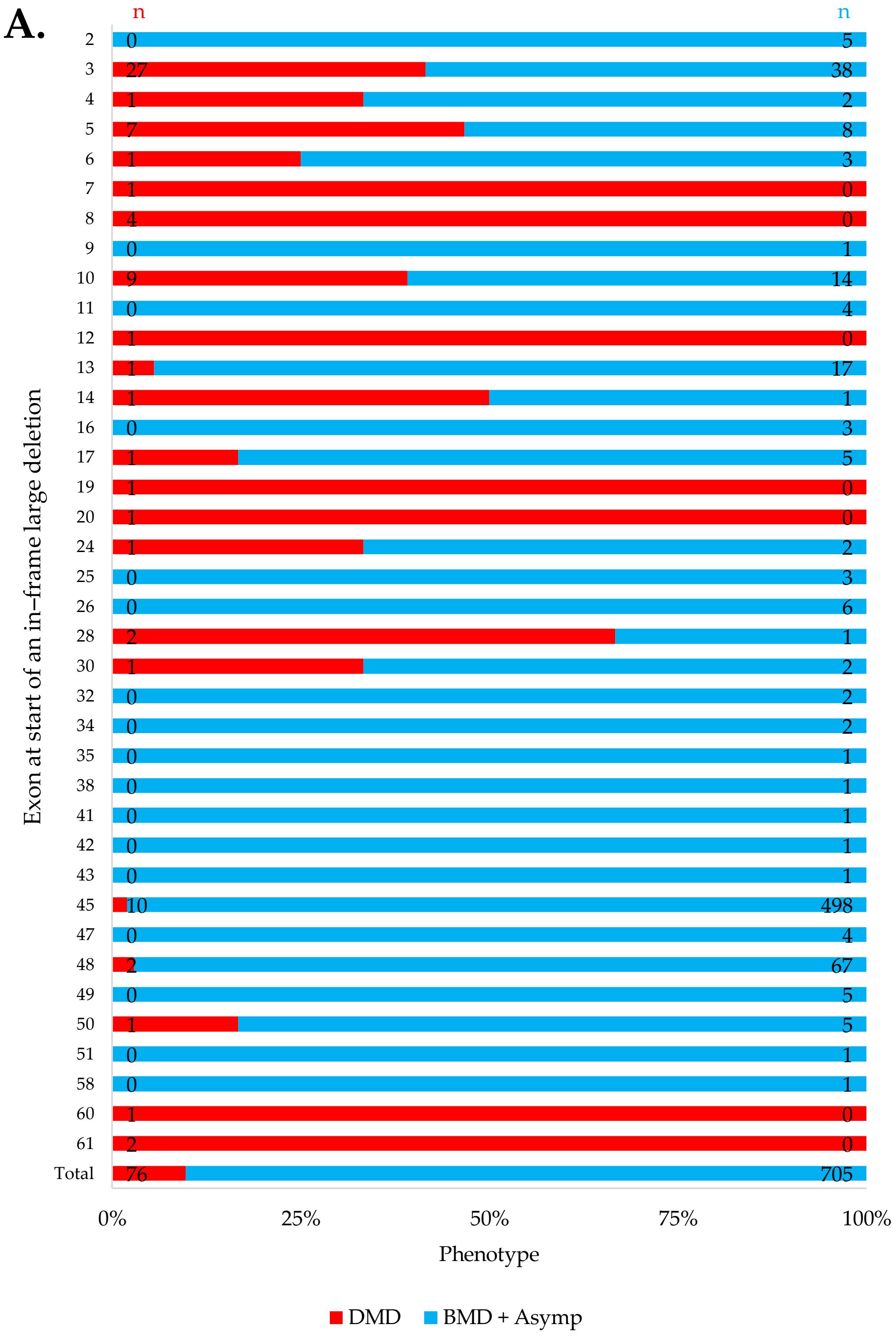

A.
n
n
Exon at start of an in-frame large deletion
2 0 5
3 27 38
4 1 2
5 7 8
6 1 3
7 1 0
8 4 0
9 0 1
10 9 14
11 0 4
12 1 0
13 1 17
14 1 1
16 0 3
17 1 5
19 1 0
20 1 0
24 1 2
25 0 3
26 0 6
28 2 1
30 1 2
32 0 2
34 0 2
35 0 1
38 0 1
41 0 1
42 0 1
43 0 1
45 10 498
47 0 4
48 2 67
49 0 5
50 1 5
51 0 1
58 0 1
60 1 0
61 2 0
Total 76 705
0%
25%
50%
75%
100%
Phenotype
DMD
BMD + Asymp

B.

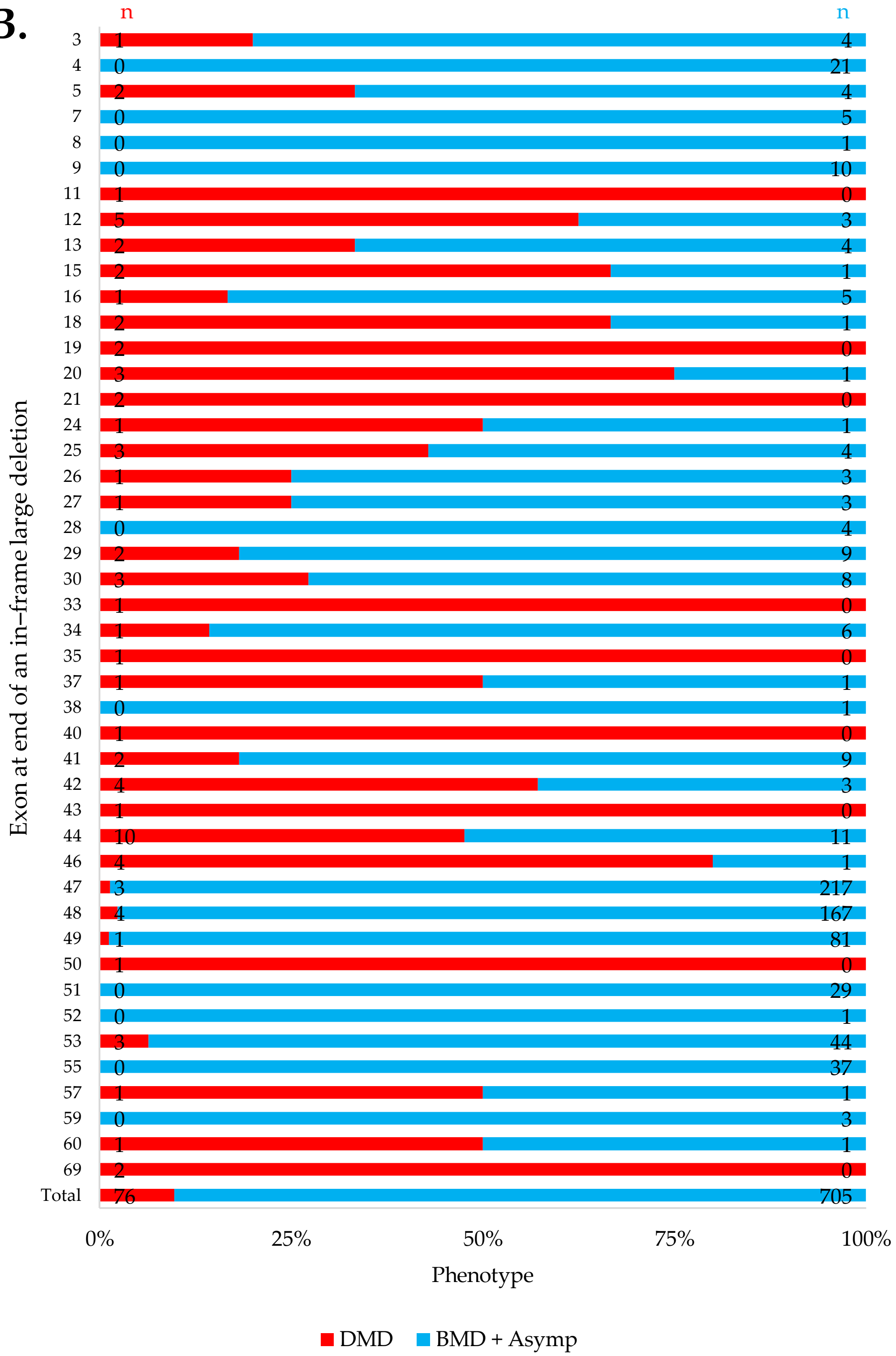
n
n
3 1 4
4 0 21
5 2 4
7 0 5
8 0 1
9 0 10
11 1 0
12 5 3
13 2 4
15 2 1
16 1 5
18 2 1
19 2 0
20 3 1
21 2 0
24 1 1
25 3 4
26 1 3
27 1 3
28 0 4
29 2 9
30 3 8
33 1 0
34 1 6
35 1 0
37 1 1
38 0 1
40 1 0
41 2 9
42 4 3
43 1 0
44 10 11
46 4 1
47 3 217
48 4 167
49 1 81
50 1 0
51 0 29
52 0 1
53 3 44
55 0 37
57 1 1
59 0 3
60 1 1
69 2 0
Total 76 705
Exon at end of an in-frame large deletion
0%
25%
50%
75%
100%
Phenotype
DMD
BMD + Asymp

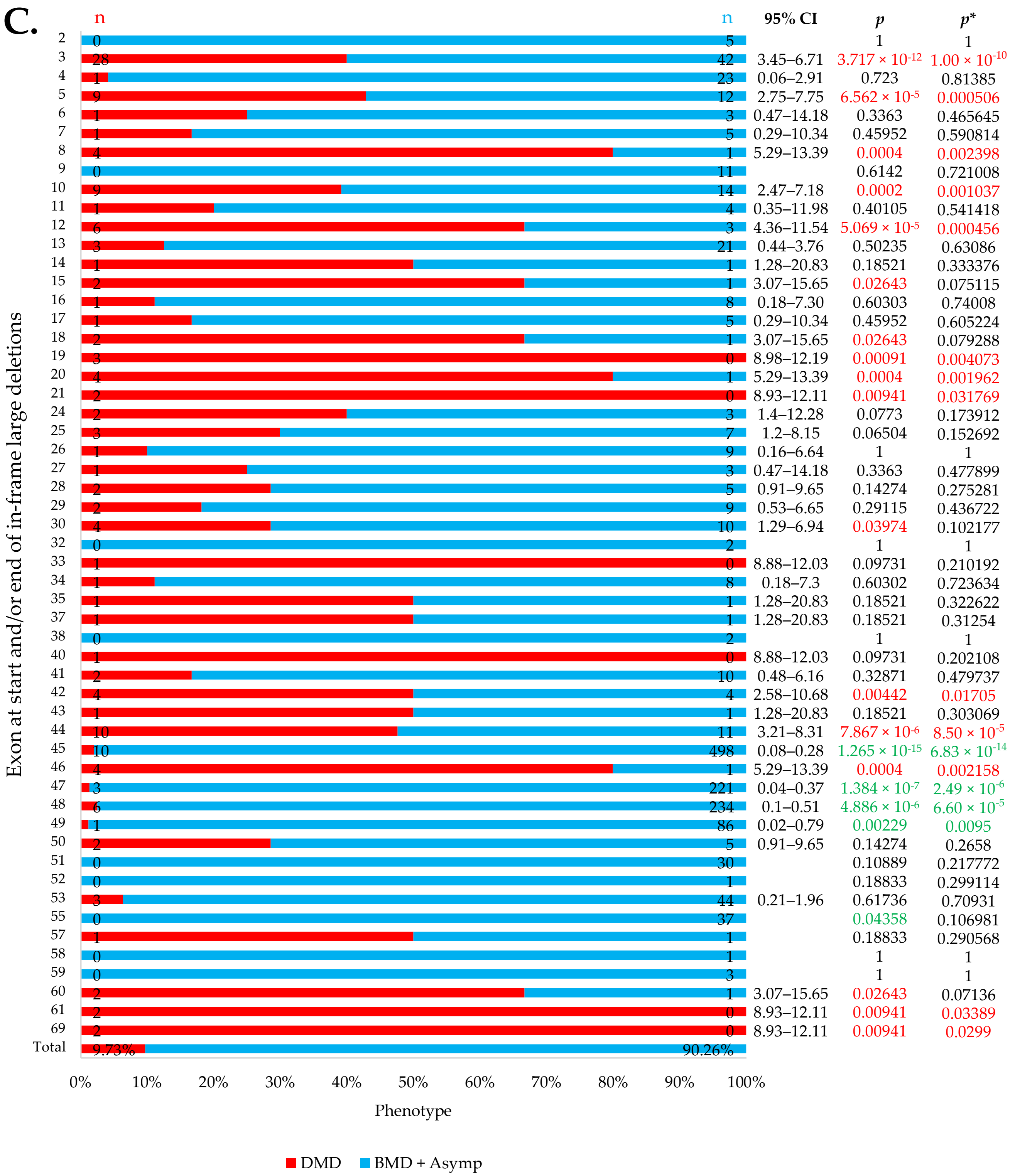

Figure S4. Association between in–frame deletions (**A**) starting, (**B**) ending, and (**C**) starting and/or ending at each exon and consequent phenotypes in the eDystrophin database. Phenotypic ratios associated with in–frame deletions starting and/or ending at a given exon and all other exons were compared using a two–tailed Fisher's exact test. n indicates the number of individuals with DMD (red) and milder (blue; BMD and asymptomatic) phenotypes. Green and red asterisks indicate a significantly lower and higher incidence of DMD phenotype, respectively. *p* = *p*-value, as calculated by Fisher's exact test; *p** = Benjamini–Hochberg adjusted *p*-value.
