# Supplementary material for: A Genotype-Phenotype Correlation Study of Exon Skip-Equivalent In-Frame Deletions and Exon Skip-Amenable Out-of-Frame Deletions across the DMD Gene to Simulate the Effects of Exon-Skipping Therapies: A Meta-Analysis
Source: J Pers Med. 2021 Jan 14;11(1):46. doi: 10.3390/jpm11010046 (PMC7830903; doi:10.3390/jpm11010046)
Supplement: Supplementary file 1 [file jpm-11-00046-s001.zip › Figure S5.pdf]

A.

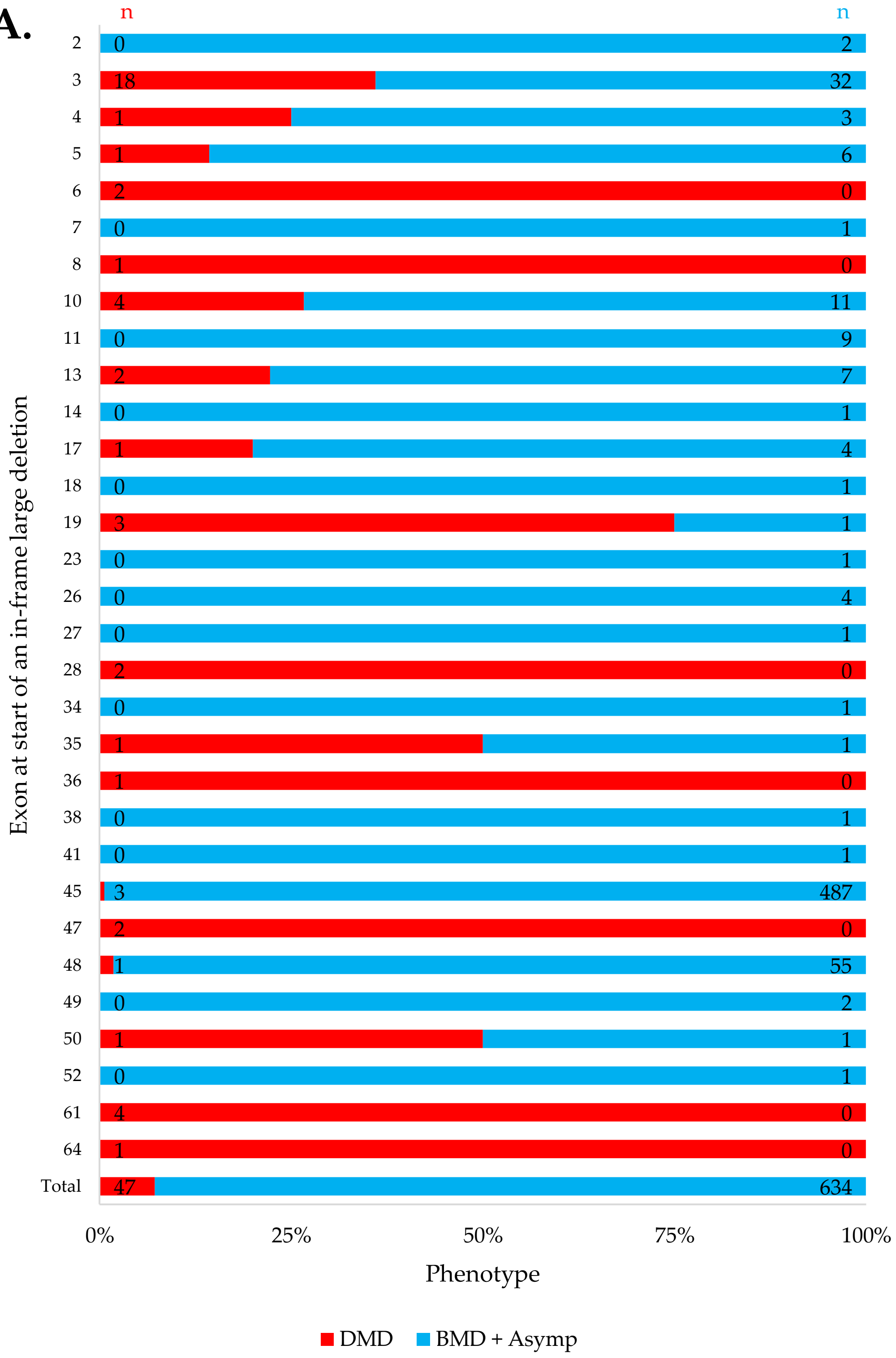
n
n
Exon at start of an in-frame large deletion
2
3
4
5
6
7
8
10
11
13
14
17
18
19
23
26
27
28
34
35
36
38
41
45
47
48
49
50
52
61
64
Total
0 2
18 32
1 3
1 6
2 0
0 1
1 0
4 11
0 9
2 7
0 1
1 4
0 1
3 1
0 1
0 4
0 1
2 0
0 1
1 1
1 0
0 1
0 1
3 487
2 0
1 55
0 2
1 1
0 1
4 0
1 0
47 634
0%
25%
50%
75%
100%
Phenotype
DMD
BMD + Asymp

B.

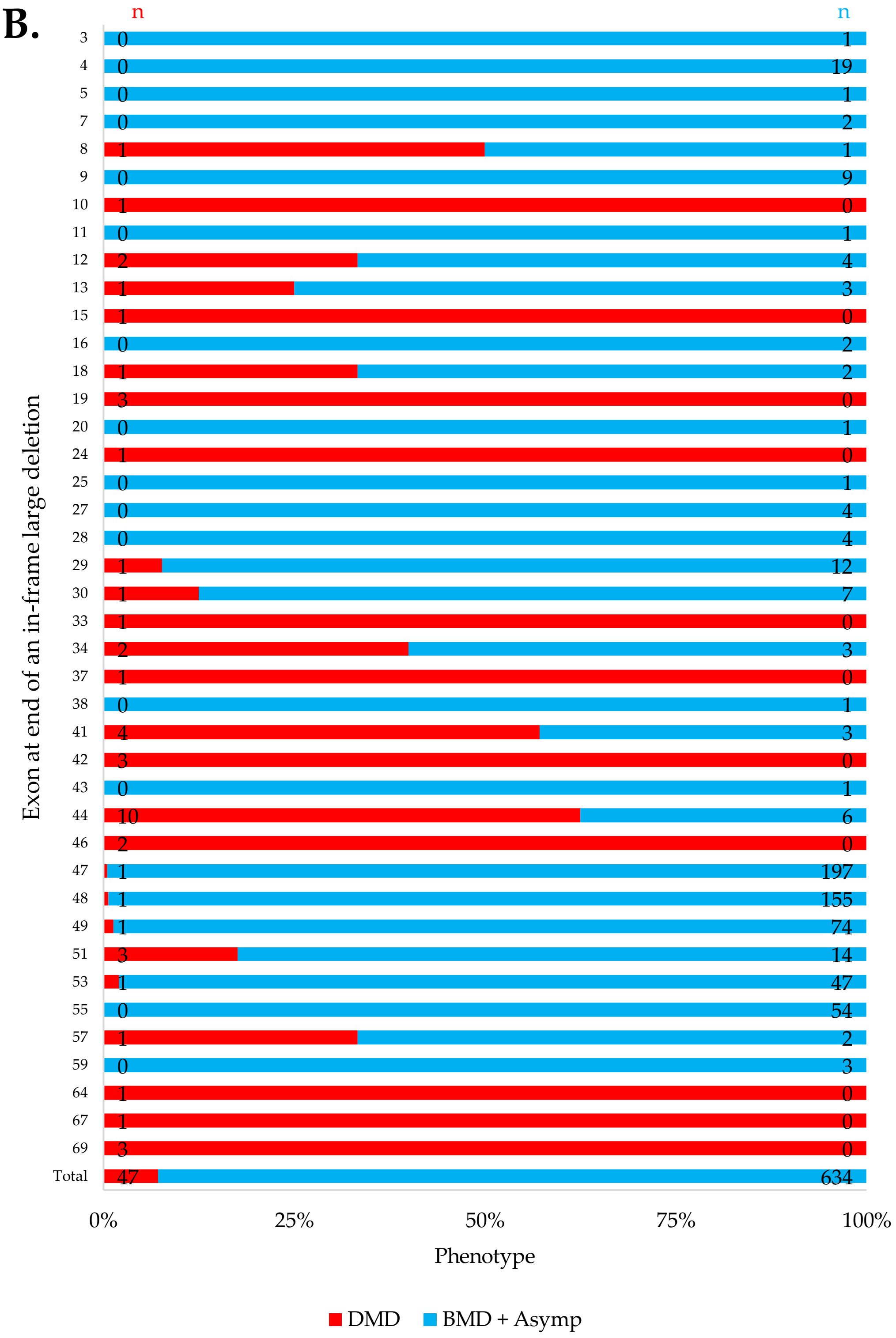

n
n
3 0 1
4 0 19
5 0 1
7 0 2
8 1 1
9 0 9
10 1 0
11 0 1
12 2 4
13 1 3
15 1 0
16 0 2
18 1 2
19 3 0
20 0 1
24 1 0
25 0 1
27 0 4
28 0 4
29 1 12
30 1 7
33 1 0
34 2 3
37 1 0
38 0 1
41 4 3
42 3 0
43 0 1
44 10 6
46 2 0
47 1 197
48 1 155
49 1 74
51 3 14
53 1 47
55 0 54
57 1 2
59 0 3
64 1 0
67 1 0
69 3 0
Total 47 634
Exon at end of an in-frame large deletion
0%
25%
50%
75%
100%
Phenotype
DMD
BMD + Asymp

C.

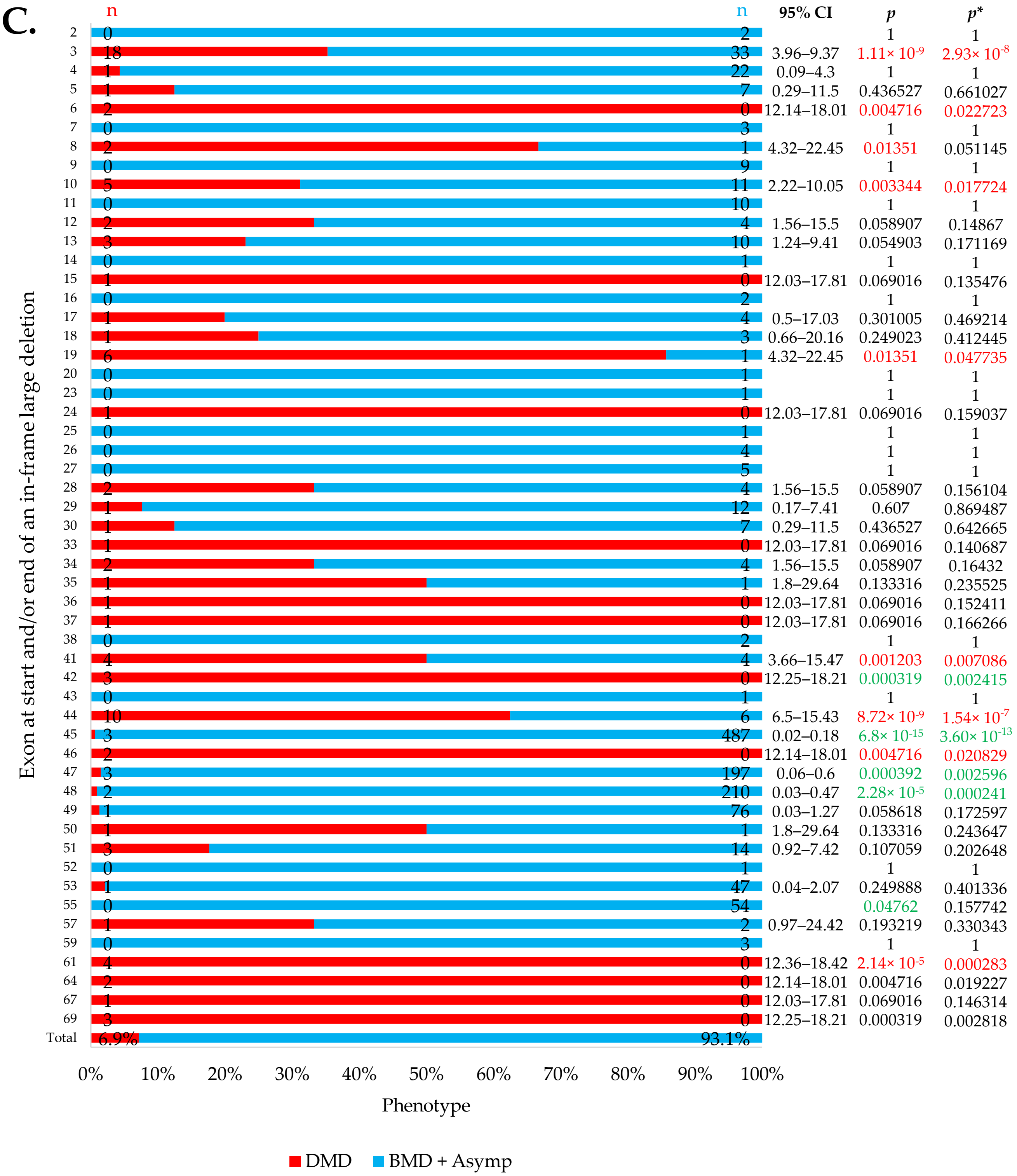

Figure S5. Association between in-frame deletions (**A**) starting, (**B**) ending, and (**C**) starting and/or ending at each exon and consequent phenotypes in the UMD-DMD France database. Phenotypic ratios associated with in-frame deletions starting and/or ending at a given exon and all other exons were compared using a two-tailed Fisher's exact test. n indicates the number of individuals with DMD (red) and milder (blue; BMD and asymptomatic) phenotypes. Green and red asterisks indicate a significantly lower and higher incidence of DMD phenotype, respectively. *p* = *p*-value, as calculated by Fisher's exact test; *p** = Benjamini-Hochberg adjusted *p*-value.
