# Supplementary material for: A Genotype-Phenotype Correlation Study of Exon Skip-Equivalent In-Frame Deletions and Exon Skip-Amenable Out-of-Frame Deletions across the DMD Gene to Simulate the Effects of Exon-Skipping Therapies: A Meta-Analysis
Source: J Pers Med. 2021 Jan 14;11(1):46. doi: 10.3390/jpm11010046 (PMC7830903; doi:10.3390/jpm11010046)
Supplement: Supplementary file 1 [file jpm-11-00046-s001.zip › Figure S6.pdf]

A.

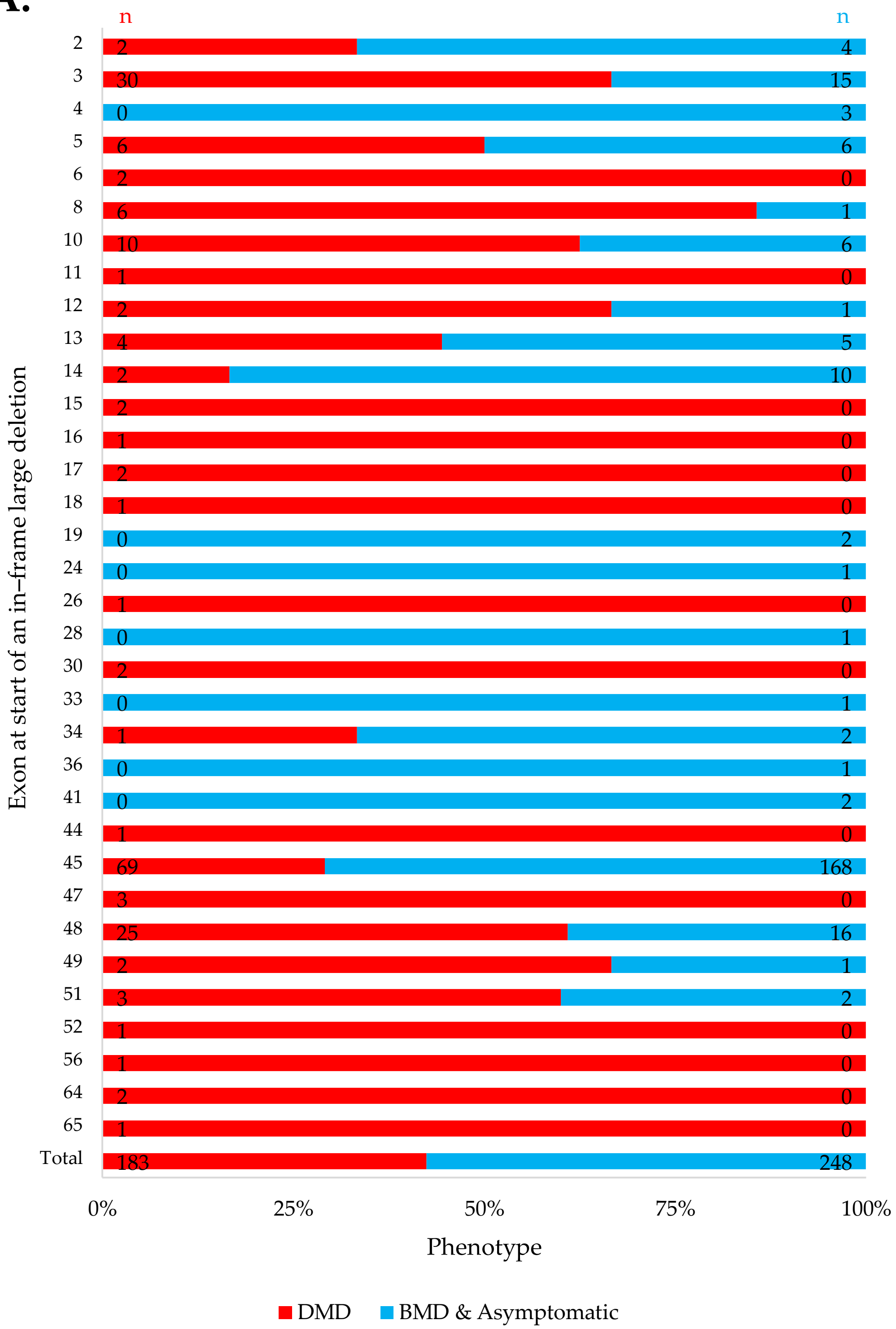

n
n
Exon at start of an in-frame large deletion
2
2
4
3
30
15
4
0
3
5
6
6
6
2
0
8
6
1
10
10
6
11
1
0
12
2
1
13
4
5
14
2
10
15
2
0
16
1
0
17
2
0
18
1
0
19
0
2
24
0
1
26
1
0
28
0
1
30
2
0
33
0
1
34
1
2
36
0
1
41
0
2
44
1
0
45
69
168
47
3
0
48
25
16
49
2
1
51
3
2
52
1
0
56
1
0
64
2
0
65
1
0
Total
183
248
0%
25%
50%
75%
100%
Phenotype
DMD
BMD & Asymptomatic

B.

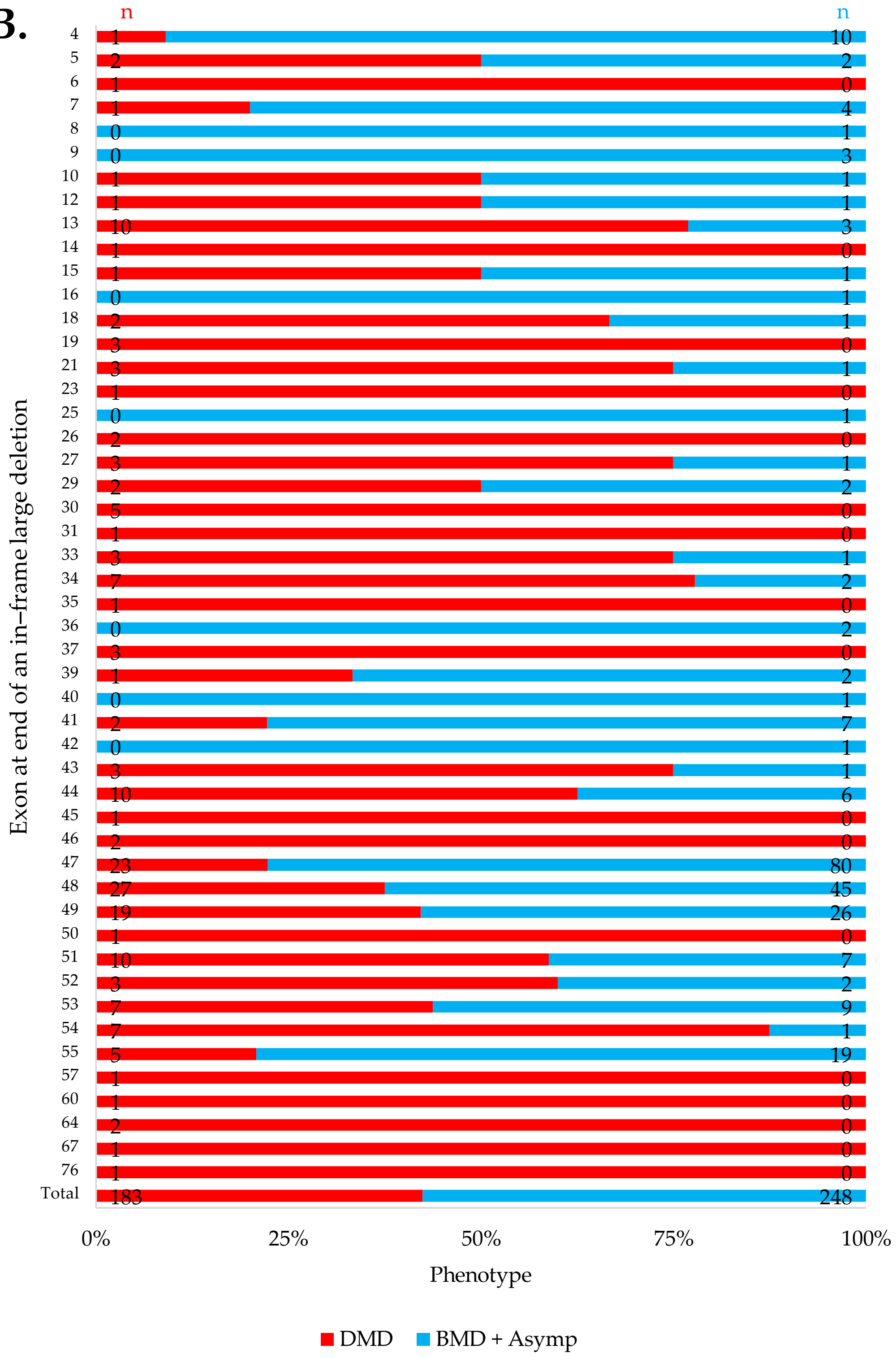
n
n
Exon at end of an in-frame large deletion
4 1 10
5 2 2
6 1 0
7 1 4
8 0 1
9 0 3
10 1 1
12 1 1
13 10 3
14 1 0
15 1 1
16 0 1
18 2 1
19 3 0
21 3 1
23 1 0
25 0 1
26 2 0
27 3 1
29 2 2
30 5 0
31 1 0
33 3 1
34 7 2
35 1 0
36 0 2
37 3 0
39 1 2
40 0 1
41 2 7
42 0 1
43 3 1
44 10 6
45 1 0
46 2 0
47 23 80
48 27 45
49 19 26
50 1 0
51 10 7
52 3 2
53 7 9
54 7 1
55 5 19
57 1 0
60 1 0
64 2 0
67 1 0
76 1 0
Total 183 248
0%
25%
50%
75%
100%
Phenotype
DMD
BMD + Asymp

C.

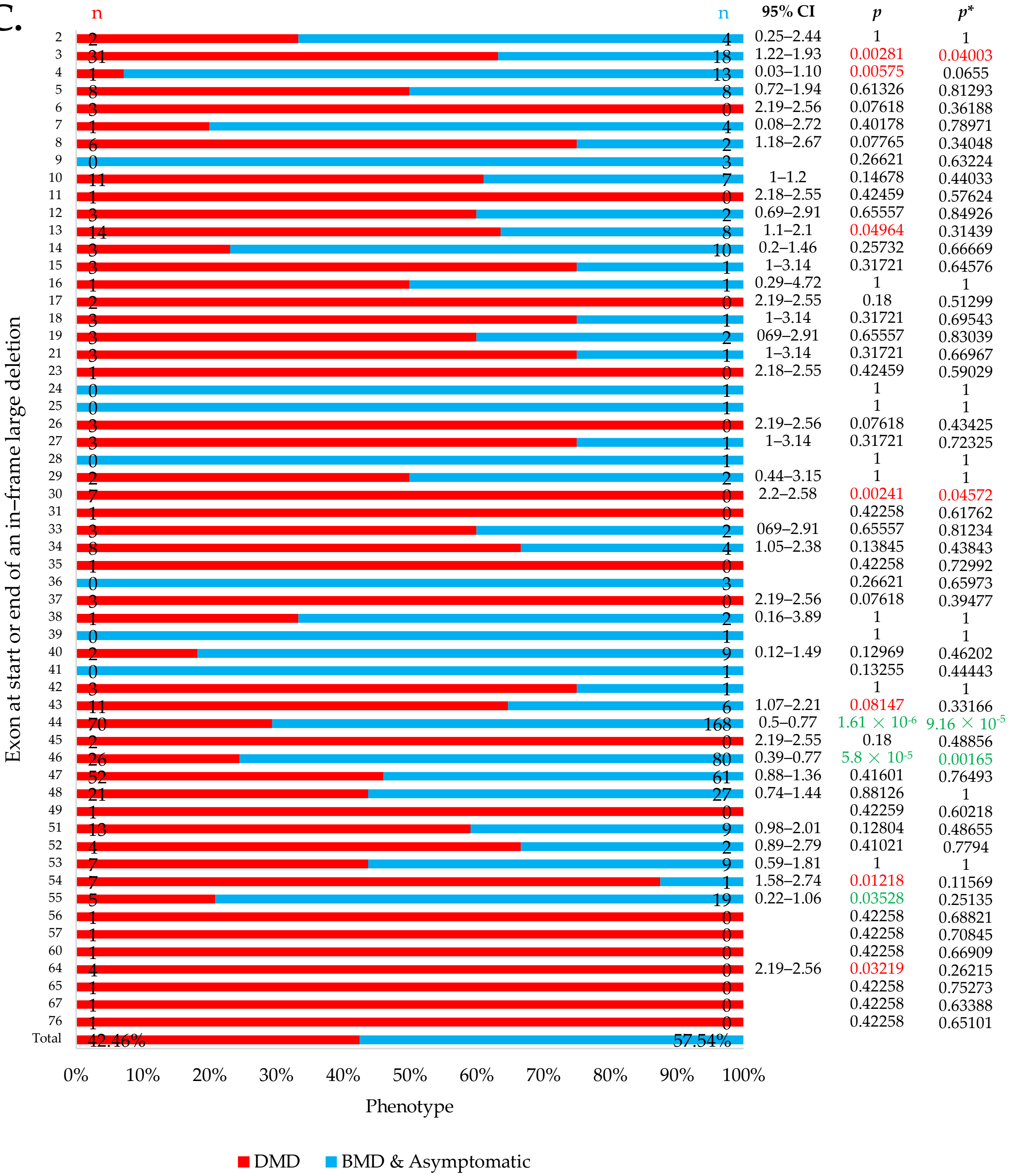

Figure S6. Association between in–frame deletions (**A**) starting, (**B**) ending, and (**C**) starting and/or ending at each exon and consequent phenotypes in the records identified from existing literature. Phenotypic ratios associated with in–frame deletions starting and/or ending at a given exon and all other exons were compared using a two–tailed Fisher's exact test. *n* indicates the number of individuals with DMD (red) and milder (blue; BMD and asymptomatic) phenotypes. Green and red asterisks indicate a significantly lower and higher incidence of DMD phenotype, respectively. *p* = *p*-value, as calculated by Fisher's exact test; *p** = Benjamini-Hochberg adjusted *p*-value.
