# Supplementary material for: A Genotype-Phenotype Correlation Study of Exon Skip-Equivalent In-Frame Deletions and Exon Skip-Amenable Out-of-Frame Deletions across the DMD Gene to Simulate the Effects of Exon-Skipping Therapies: A Meta-Analysis
Source: J Pers Med. 2021 Jan 14;11(1):46. doi: 10.3390/jpm11010046 (PMC7830903; doi:10.3390/jpm11010046)
Supplement: Supplementary file 1 [file jpm-11-00046-s001.zip › Figure S7.pdf]

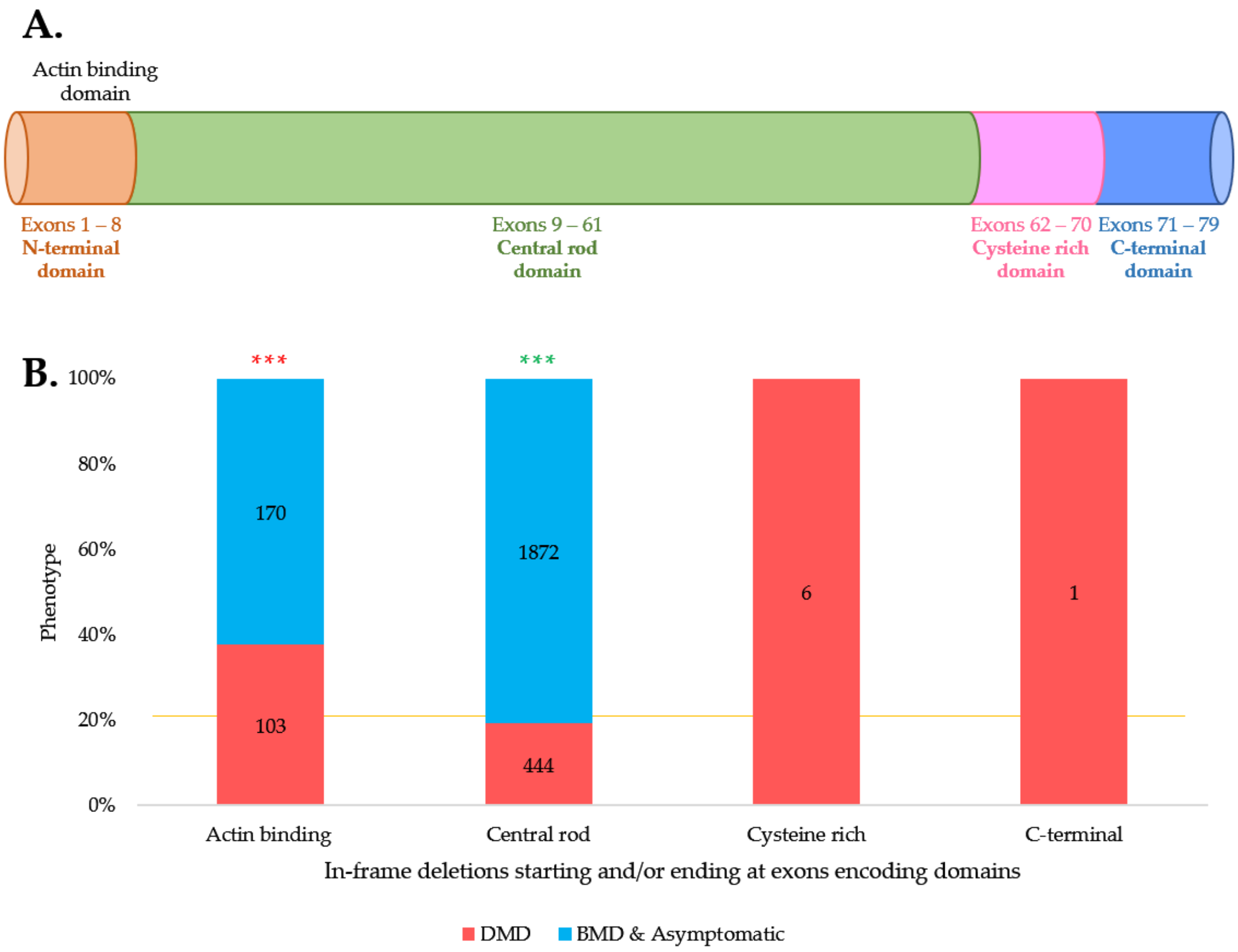

Figure S7. Overview of clinical phenotypes associated with in-frame deletions starting and/or ending at different exons and their corresponding dystrophin domains. **(A)** Schematic illustration of dystrophin of domains and the exons encoding those domains [1]. **(B)** Clinical phenotypes of in-frame deletions starting and/or ending at exons encoding different domains of dystrophin. Green and red asterisks indicate a significantly lower and higher incidence of DMD phenotype for a given domain, respectively, as compared to the overall incidence rate. Data on the in-frame large deletions' phenotypic outcomes were collected from the UMD-DMD France Knowledgebase, eDystrophin database, and the existing literature. The yellow line indicates the overall incidence rate of DMD phenotype (**Figure 2**). We compared the phenotypic ratios associated with in-frame deletions starting and/or ending at the exons coding for a given domain and all other exons. The statistical significance was determined using a two-tailed Fisher's Exact test. (*** = $p < 0.0001$).

References:

1. Le Rumeur, E. Dystrophin and the two related genetic diseases, Duchenne and Becker muscular dystrophies. *Bosn. J. Basic Med. Sci*. 2015, 15, doi:10.17305/bjbms.2015.636.
