# Supplementary material for: A Genotype-Phenotype Correlation Study of Exon Skip-Equivalent In-Frame Deletions and Exon Skip-Amenable Out-of-Frame Deletions across the DMD Gene to Simulate the Effects of Exon-Skipping Therapies: A Meta-Analysis
Source: J Pers Med. 2021 Jan 14;11(1):46. doi: 10.3390/jpm11010046 (PMC7830903; doi:10.3390/jpm11010046)
Supplement: Supplementary file 1 [file jpm-11-00046-s001.zip › Figure S8.pdf]

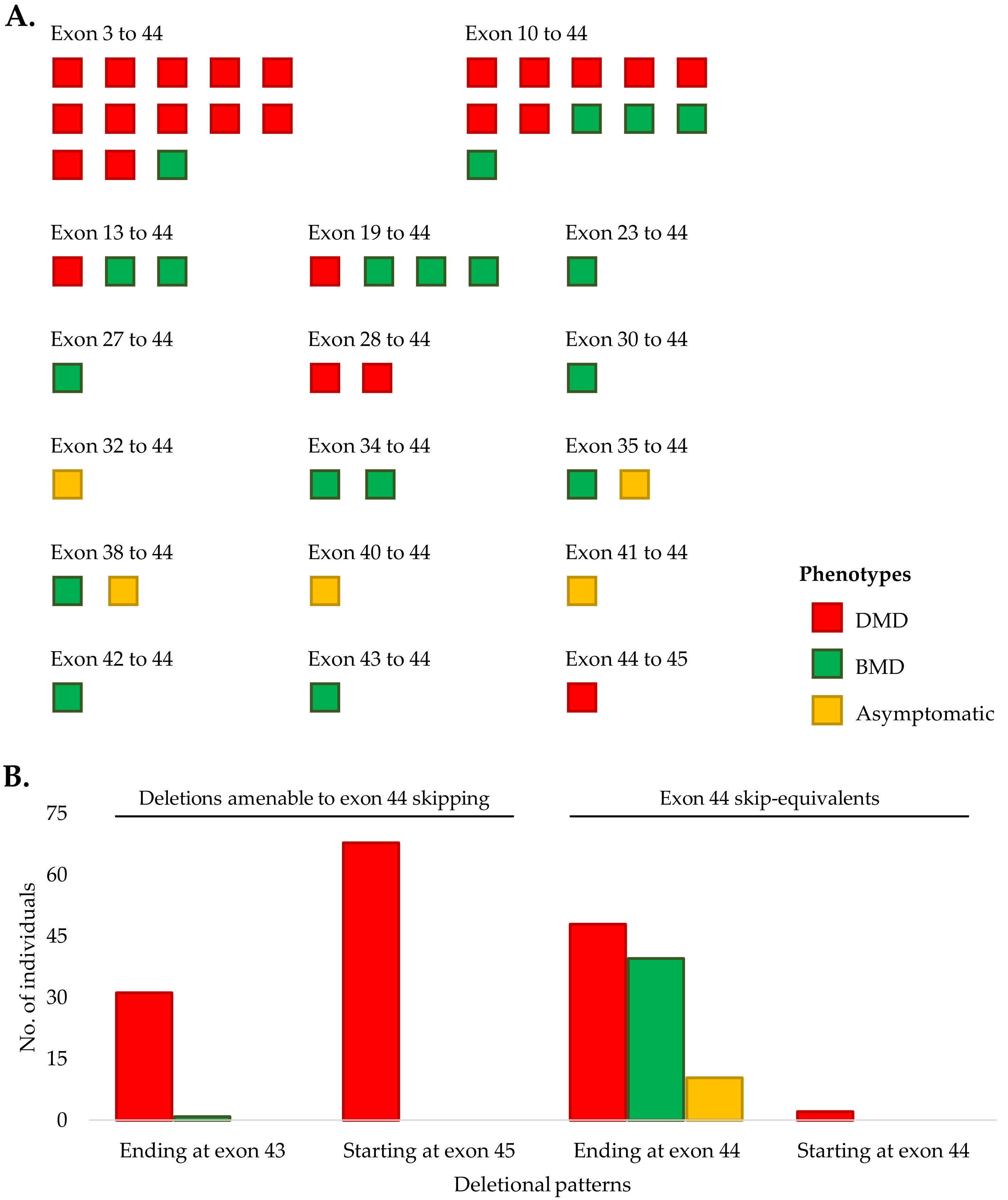

Figure S8: Phenotypes associated with different exon 44 skip equivalent and amenable to exon 44 skipping mutations. (**A**) Distribution of phenotypes for each exon-44 skip equivalent deletion subset. Individual patients are grouped depending on the pattern of deletion and their phenotypes are represented by colored squares. (**B**) Phenotypic comparison of deletional patterns between in-frame skip-equivalent deletions and patients eligible for exon 44 skipping with out-of-frame deletions (UMD-DMD France database).
