# Supplementary material for: A Genotype-Phenotype Correlation Study of Exon Skip-Equivalent In-Frame Deletions and Exon Skip-Amenable Out-of-Frame Deletions across the DMD Gene to Simulate the Effects of Exon-Skipping Therapies: A Meta-Analysis
Source: J Pers Med. 2021 Jan 14;11(1):46. doi: 10.3390/jpm11010046 (PMC7830903; doi:10.3390/jpm11010046)
Supplement: Supplementary file 1 [file jpm-11-00046-s001.zip › Table S1.pdf]

Table S1: Clinical data obtained from literature searching.

In-frame deletion starts at exon	In-frame deletion ends at exon	Clinical phenotype		References
		DMD	BMD & Asymptomatic	
2	7	1	4	[1,2]
2	50	1		[3]
3	3	1	3	[1,4,5]
3	4	1	7	[2,3,5,6]
3	5		1	[6]
3	6	1		[3]
3	8		1	[7]
3	10	1		[8]
3	13	5		[2,8,9]
3	18	2		[3]
3	26	2		[8]
3	27	2		[8]
3	29	1	1	[8]
3	30	1		[8]
3	33	1		[8]
3	34	1		[7]
3	35	1		[7]
3	37	3		[3,7,8]
3	39		1	[10]
3	44	7		[2,3,7,8]
4	4		3	[2,4,6]
5	5	2	1	[2–4]
5	9		3	[6,10]
5	13	2		[2]
5	15		1	[10]
5	16		1	[2]
5	27	1		[7]
5	33	1		[8]
6	33	1		[3]
6	48	1		[8]
8	19	3		[1,3,9]
8	21	3	1	[8]
10	10		1	[6]
10	12	1		[1]
10	13	2		[3,10]
10	25		1	[6]

10	30	1		[8]
10	33		1	[6]
10	34	2	1	[3,6,8]
10	41	1		[8]
10	42		1	[6]
10	44	3	1	[3,6,8]
11	39	1		[7]
12	43	2	1	[5,6,8]
13	13	1	3	[4,6,7]
13	18		1	[6]
13	34	1		[8]
13	41	1		[7]
13	44		1	[6]
13	47	1		[2]
14	14	1		[8]
14	27		1	[10]
14	30	1		[3]
14	36		1	[10]
14	39		1	[2]
14	40		1	[2]
14	41		5	[6]
14	51		1	[10]
15	15	1		[3]
15	29	1		[3]
16	23	1		[3]
17	30	1		[8]
17	34	1		[7]
18	43	1		[7]
19	44		2	[6]
24	48		1	[6]
26	34	1		[8]
28	29		1	[4]
30	30	1		[3]
30	31	1		[3]
33	34		1	[4]
34	34	1		[8]
34	44		2	[6]
36	36		1	[4]
41	41		2	[4]
44	45	1		[8]
45	46	2		[2,8]

45	47	21	80	[2,3,5–8,11,12]
45	48	17	40	[2,3,6–8,11,12]
45	49	11	18	[2,3,6,8,12]
45	51	1	3	[2,6,12]
45	53	6	9	[2,3,6–8]
45	55	3	17	[2,3,6,8]
45	54	7	1	[3,6]
45	57	1		[3]
47	47	1		[3]
47	48	1		[2]
47	51	1		[5]
48	48	8	4	[2,3,6–8]
48	49	8	8	[2,3,7,8,12]
48	51	6	2	[2,3,8,12]
48	55	2	2	[6,7]
48	67	1		[8]
49	51	2	1	[2,3,9]
51	52	3	2	[8]
52	53	1		[5]
56	60	1		[3]
64	64	2		[7,8]
65	76	1		[5]
	**Total**	183	248	

References:

1. Vieitez, I.; Gallano, P.; González-Quereda, L.; Borrego, S.; Marcos, I.; Millán, J.M.; Jairo, T.; Prior, C.; Molano, J.; Trujillo-Tiebas, M.J.; et al. Mutational spectrum of Duchenne muscular dystrophy in Spain: study of 284 cases. Neurología 2017, 32, 377–385, doi:10.1016/j.nrleng.2015.12.004.
2. Mah, J.K.; Selby, K.; Campbell, C.; Nadeau, A.; Tarnopolsky, M.; McCormick, A.; Dooley, J.M.; Kolski, H.; Skalsky, A.J.; Smith, R.G.; et al. A population-based study of dystrophin mutations in Canada. Can. J. Neurol. Sci. 2011, 38, 465–74, doi:10.1017/s0317167100011896.
3. Guo, R.; Zhu, G.; Zhu, H.; Ma, R.; Peng, Y.; Liang, D.; Wu, L. DMD mutation spectrum analysis in 613 Chinese patients with dystrophinopathy. J. Hum. Genet. 2015, 60, 435–442, doi:10.1038/jhg.2015.43.
4. Deburgrave, N.; Daoud, F.; Llense, S.; Barbot, J.C.; Récan, D.; Peccate, C.; Burghes, A.H.M.; Béroud, C.; Garcia, L.; Kaplan, J.-C.; et al. Protein- and mRNA-based phenotype-genotype correlations in DMD/BMD with point mutations and molecular basis for BMD with nonsense and frameshift mutations in the DMD gene. Hum. Mutat. 2007, 28, 183–195, doi:10.1002/humu.20422.
5. Fain-man Lo, I.; Lai, K.K.; Tong, T.M.; Lam, S.T. A different spectrum of DMD gene mutations in local Chinese patients with Duchenne/Becker muscular dystrophy. Chin. Med. J. (Engl). 2006, 119, 1079–87.
6. Mori-Yoshimura, M.; Mitsuhashi, S.; Nakamura, H.; Komaki, H.; Goto, K.; Yonemoto, N.; Takeuchi, F.; Hayashi, Y.K.; Murata, M.; Takahashi, Y.; et al. Characteristics of Japanese Patients with Becker Muscular

Dystrophy and Intermediate Muscular Dystrophy in a Japanese National Registry of Muscular Dystrophy (Remudy): Heterogeneity and Clinical Variation. J. Neuromuscul. Dis. 2018, 5, 193–203, doi:10.3233/JND-170225.
7. Tran, V.K.; Ta, V.T.; Vu, D.C.; Nguyen, S.T.-B.; Do, H.N.; Ta, M.H.; Tran, T.H.; Matsuo, M. Exon Deletion Patterns of the Dystrophin Gene in 82 Vietnamese Duchenne/Becker Muscular Dystrophy Patients. J. Neurogenet. 2013, 27, 170–175, doi:10.3109/01677063.2013.830616.
8. Yang, J.; Li, S.Y.; Li, Y.Q.; Cao, J.Q.; Feng, S.W.; Wang, Y.Y.; Zhan, Y.X.; Yu, C.S.; Chen, F.; Li, J.; et al. MLPA-based genotype–phenotype analysis in 1053 Chinese patients with DMD/BMD. BMC Med. Genet. 2013, 14, 29, doi:10.1186/1471-2350-14-29.
9. Rani, A.Q.; Sasongko, T.H.; Sulong, S.; Bunyan, D.; Salmi, A.R.; Zilfalil, B.A.; Matsuo, M.; Zabidi-Hussin, Z.A.M.H. Mutation spectrum of dystrophin gene in malaysian patients with Duchenne/Becker muscular dystrophy. J. Neurogenet. 2013, 27, 11–5, doi:10.3109/01677063.2012.762580.
10. Ji, X.; Zhang, J.; Xu, Y.; Long, F.; Sun, W.; Liu, X.; Chen, Y.; Jiang, W. MLPA Application in Clinical Diagnosis of DMD/BMD in Shanghai. J. Clin. Lab. Anal. 2015, 29, 405–411, doi:10.1002/jcla.21787.
11. Todorova, A.; Todorov, T.; Georgieva, B.; Lukova, M.; Guergueltcheva, V.; Kremensky, I.; Mitev, V. MLPA analysis/complete sequencing of the DMD gene in a group of Bulgarian Duchenne/Becker muscular dystrophy patients. Neuromuscul. Disord. 2008, 18, 667–70, doi:10.1016/j.nmd.2008.06.369.
12. Li, Y.; Liu, Z.; OuYang, S.; Zhu, Y.; Wang, L.; Wu, J. Distribution of dystrophin gene deletions in a Chinese population. J. Int. Med. Res. 2016, 44, 99–108, doi:10.1177/0300060515613223.
