# Supplementary material for: A Genotype-Phenotype Correlation Study of Exon Skip-Equivalent In-Frame Deletions and Exon Skip-Amenable Out-of-Frame Deletions across the DMD Gene to Simulate the Effects of Exon-Skipping Therapies: A Meta-Analysis
Source: J Pers Med. 2021 Jan 14;11(1):46. doi: 10.3390/jpm11010046 (PMC7830903; doi:10.3390/jpm11010046)
Supplement: Supplementary file 1 [file jpm-11-00046-s001.zip › Table S2.pdf]

Table S2: List of all theoretically possible in-frame deletion within *dystrophin*. Bold-underlined numbers indicate deletions found in the eDystrophin database, while cells with green fonts and yellow highlighter refers to the deletions obtained from the UMD-DMD France database and literature searching, respectively.

Deletion starts at exon	Deletion ends at exon	Deletion starts at exon	Deletion ends at exon
2	7	23	57
2	19	23	59
2	21	23	60
2	50	23	67
2	52	23	69
2	58	23	75
2	61	23	78
2	63	**24**	**25**?
2	64	**24**	**25**
2	66	24	26
2	76	**24**	**27**
2	77	24	28
3	3	24	29
3	4	24	30
3	5	24	31
3	8	24	32
3	9	24	33
3	10	24	34
3	12	24	35
3	13	24	36
3	14	24	37
3	15	24	38
3	16	24	39
3	18	24	40
3	20	24	41
3	22	24	42
3	23	24	44
3	24	24	46
3	25	24	47
**3**	**26**	24	48
3	27	24	49
3	28	24	51
3	29	24	53
3	30	24	55
3	31	24	57
3	32	24	59
3	33	24	60
3	34	24	67

Deletion starts at exon	Deletion ends at exon	Deletion starts at exon	Deletion ends at exon
**3**	**35**	24	69
3	36	24	75
3	37	24	78
3	38	**25**	**25**
3	39	25	26
3	40	25	27
3	41	25	28
**3**	**42**	25	29
3	44	25	30
3	46	25	31
**3**	**47**	25	32
3	48	25	33
3	49	25	34
3	51	25	35
3	53	25	36
3	55	25	37
3	57	25	38
3	59	25	39
3	60	25	40
3	67	25	41
3	69	25	42
3	75	25	44
3	78	25	46
4	4	25	47
4	5	25	48
4	8	25	49
4	9	25	51
4	10	25	53
4	12	25	55
4	13	25	57
4	14	25	59
4	15	25	60
4	16	25	67
4	18	25	69
4	20	25	75
4	22	25	78
4	23	**26**	**26**
4	24	26	27
4	25	26	28
4	26	26	29
4	27	26	30
4	28	26	31

Deletion starts at exon	Deletion ends at exon	Deletion starts at exon	Deletion ends at exon
4	29	26	32
**4**	**30**	26	33
4	31	26	34
4	32	26	35
4	33	26	36
4	34	26	37
4	35	26	38
4	36	26	39
4	37	26	40
4	38	26	41
4	39	26	42
4	40	26	44
4	41	26	46
4	42	26	47
4	44	26	48
4	46	26	49
4	47	26	51
4	48	26	53
4	49	26	55
4	51	26	57
4	53	26	59
4	55	26	60
4	57	26	67
4	59	26	69
4	60	26	75
4	67	26	78
4	69	27	27
4	75	27	28
4	78	27	29
**5**	**5**	27	30
5	8	27	31
**5**	**9**	27	32
5	10	27	33
5	12	27	34
**5**	**13**	27	35
5	14	27	36
5	15	27	37
5	16	27	38
**5**	**18**	27	39
5	20	27	40
5	22	27	41
5	23	27	42

Deletion starts at exon	Deletion ends at exon	Deletion starts at exon	Deletion ends at exon
5	24	27	44
5	25	27	46
5	26	27	47
5	27	27	48
5	28	27	49
5	29	27	51
5	30	27	53
5	31	27	55
5	32	27	57
5	33	27	59
5	34	27	60
5	35	27	67
5	36	27	69
**5**	**37**	27	75
5	38	27	78
5	39	28	28
5	40	**28**	**29**
5	41	28	30
5	42	28	31
**5**	**44**	28	32
5	46	28	33
5	47	28	34
5	48	28	35
5	49	28	36
5	51	28	37
5	53	28	38
5	55	28	39
5	57	28	40
5	59	28	41
5	60	28	42
5	67	28	44
5	69	28	46
5	75	28	47
5	78	28	48
**6**	**8**	**28**	**49**
6	9	28	51
6	10	28	53
6	12	28	55
6	13	28	57
6	14	28	59
6	15	28	60
6	16	28	67

Deletion starts at exon	Deletion ends at exon	Deletion starts at exon	Deletion ends at exon
6	18	28	69
6	20	28	75
6	22	28	78
6	23	29	29
6	24	29	30
6	25	29	31
6	26	29	32
6	27	29	33
6	28	29	34
6	29	29	35
6	30	29	36
6	31	29	37
6	32	29	38
6	33	29	39
6	34	29	40
6	35	29	41
6	36	29	42
6	37	29	44
6	38	29	46
6	39	29	47
6	40	29	48
6	41	29	49
6	42	29	51
6	44	29	53
6	46	29	55
6	47	29	57
6	48	29	59
6	49	29	60
6	51	29	67
6	53	29	69
6	55	29	75
6	57	29	78
6	59	**30**	**30**
6	60	30	31
6	67	30	32
6	69	30	33
6	75	30	34
6	78	30	35
7	11	30	36
7	17	30	37
7	43	30	38
7	45	30	39

Deletion starts at exon	Deletion ends at exon	Deletion starts at exon	Deletion ends at exon
7	54	30	40
7	56	30	41
7	62	**30**	**42**
7	65	**30**	**44**
7	68	30	46
7	70	30	47
7	71	30	48
7	72	30	49
7	73	30	51
7	74	30	53
8	19	30	55
**8**	**21**	30	57
8	50	30	59
8	52	30	60
8	58	30	67
8	61	30	69
8	63	30	75
8	64	30	78
8	66	31	31
8	76	31	32
8	77	31	33
9	9	31	34
9	10	31	35
**9**	**12**	31	36
9	13	31	37
9	14	31	38
9	15	31	39
9	16	31	40
9	18	31	41
9	20	31	42
9	22	31	44
9	23	31	46
9	24	31	47
9	25	31	48
9	26	31	49
9	27	31	51
9	28	31	53
9	29	31	55
9	30	31	57
9	31	31	59
9	32	31	60
9	33	31	67

Deletion starts at exon	Deletion ends at exon	Deletion starts at exon	Deletion ends at exon
9	34	31	69
9	35	31	75
9	36	31	78
9	37	32	32
9	38	32	33
9	39	32	34
9	40	32	35
9	41	32	36
9	42	32	37
9	44	32	38
9	46	32	39
9	47	32	40
9	48	32	41
9	49	**32**	**42**
9	51	**32**	**44**
9	53	32	46
9	55	32	47
9	57	32	48
9	59	32	49
9	60	32	51
9	67	32	53
9	69	32	55
9	75	32	57
9	78	32	59
10	10	32	60
**10**	**12**	32	67
**10**	**13**	32	69
10	14	32	75
**10**	**15**	32	78
**10**	**16**	33	33
**10**	**18**	33	34
10	20	33	35
10	22	33	36
10	23	33	37
10	24	33	38
10	25	33	39
**10**	**26**	33	40
10	27	33	41
**10**	**28**	33	42
**10**	**29**	33	44
10	30	33	46
10	31	33	47

Deletion starts at exon	Deletion ends at exon	Deletion starts at exon	Deletion ends at exon
10	32	33	48
10	33	33	49
10	34	33	51
10	35	33	53
10	36	33	55
**10**	**37**	33	57
10	38	33	59
10	39	33	60
10	40	33	67
10	41	33	69
**10**	**42**	33	75
**10**	**44**	33	78
10	46	34	34
10	47	34	35
**10**	**48**	34	36
10	49	34	37
10	51	34	38
10	53	34	39
10	55	34	40
10	57	34	41
10	59	34	42
10	60	34	44
10	67	34	46
10	69	34	47
10	75	34	48
10	78	34	49
11	12	34	51
11	13	34	53
11	14	34	55
11	15	34	57
11	16	34	59
11	18	34	60
11	20	34	67
11	22	34	69
11	23	34	75
11	24	34	78
11	25	35	35
11	26	35	36
**11**	**27**	35	37
11	28	35	38
**11**	**29**	35	39
**11**	**30**	35	40

Deletion starts at exon	Deletion ends at exon	Deletion starts at exon	Deletion ends at exon
11	31	35	41
11	32	35	42
11	33	35	44
11	34	35	46
11	35	35	47
11	36	35	48
11	37	35	49
11	38	35	51
11	39	35	53
11	40	35	55
11	41	35	57
11	42	35	59
11	44	35	60
11	46	35	67
11	47	35	69
11	48	35	75
11	49	35	78
11	51	36	36
11	53	36	37
11	55	36	38
11	57	36	39
11	59	36	40
11	60	36	41
11	67	36	42
11	69	36	44
11	75	36	46
11	78	36	47
12	17	36	48
12	43	36	49
12	45	36	51
12	54	36	53
12	56	36	55
12	62	36	57
12	65	36	59
12	68	36	60
12	70	36	67
12	71	36	69
12	72	36	75
12	73	36	78
12	74	37	37
13	13	37	38
13	14	37	39

Deletion starts at exon	Deletion ends at exon	Deletion starts at exon	Deletion ends at exon
13	15	37	40
13	16	37	41
13	18	37	42
13	20	37	44
13	22	37	46
13	23	37	47
13	24	37	48
13	25	37	49
13	26	37	51
13	27	37	53
13	28	37	55
**13**	**29**	37	57
**13**	**30**	37	59
13	31	37	60
13	32	37	67
13	33	37	69
**13**	**34**	37	75
13	35	37	78
13	36	38	38
**13**	**37**	38	39
13	38	38	40
13	39	38	41
**13**	**40**	38	42
**13**	**41**	**38**	**44**
**13**	**42**	38	46
**13**	**44**	38	47
13	46	38	48
13	47	38	49
13	48	38	51
13	49	38	53
13	51	38	55
13	53	38	57
13	55	38	59
13	57	38	60
13	59	38	67
13	60	38	69
13	67	38	75
13	69	38	78
13	75	39	39
13	78	39	40
14	14	39	41
**14**	**15**	39	42

Deletion starts at exon	Deletion ends at exon	Deletion starts at exon	Deletion ends at exon
14	16	39	44
14	18	39	46
14	20	39	47
14	22	39	48
14	23	39	49
14	24	39	51
14	25	39	53
14	26	39	55
14	27	39	57
14	28	39	59
14	29	39	60
14	30	39	67
14	31	39	69
14	32	39	75
14	33	39	78
14	34	40	40
14	35	40	41
14	36	40	42
14	37	**40**	**44**
14	38	40	46
14	39	40	47
14	40	40	48
14	41	40	49
14	42	40	51
14	44	40	53
14	46	40	55
14	47	40	57
14	48	40	59
14	49	40	60
14	51	40	67
14	53	40	69
14	55	40	75
14	57	40	78
14	59	41	41
14	60	41	42
14	67	**41**	**44**
14	69	41	46
14	75	41	47
14	78	41	48
15	15	41	49
15	16	41	51
15	18	41	53

Deletion starts at exon	Deletion ends at exon	Deletion starts at exon	Deletion ends at exon
15	20	41	55
15	22	41	57
15	23	41	59
15	24	41	60
15	25	41	67
15	26	41	69
15	27	41	75
15	28	41	78
15	29	42	42
15	30	**42**	**44**
15	31	42	46
15	32	42	47
15	33	42	48
15	34	42	49
15	35	42	51
15	36	42	53
15	37	42	55
15	38	42	57
15	39	42	59
15	40	42	60
15	41	42	67
15	42	42	69
15	44	42	75
15	46	42	78
15	47	**43**	**44**
15	48	43	46
15	49	43	47
15	51	43	48
15	53	43	49
15	55	43	51
15	57	43	53
15	59	43	55
15	60	43	57
15	67	43	59
15	69	43	60
15	75	43	67
15	78	43	69
**16**	**16**	43	75
16	18	43	78
16	20	**44**	**45**
16	22	**44**	**54**
16	23	44	56

Deletion starts at exon	Deletion ends at exon	Deletion starts at exon	Deletion ends at exon
16	24	44	62
16	25	44	65
16	26	44	68
16	27	44	70
16	28	44	71
**16**	**29**	44	72
16	30	44	73
16	31	44	74
16	32	**45**	**46**
16	33	**45**	**47**
16	34	**45**	**48**
16	35	**45**	**49**
16	36	**45**	**51**
16	37	**45**	**53**
16	38	**45**	**55**
16	39	**45**	**57**
16	40	45	59
16	41	45	60
16	42	45	67
16	44	45	69
16	46	45	75
16	47	45	78
16	48	46	54
16	49	46	56
16	51	46	62
16	53	46	65
16	55	46	68
16	57	46	70
16	59	46	71
16	60	46	72
16	67	46	73
16	69	46	74
16	75	**47**	**47**
16	78	**47**	**48**
17	18	47	49
17	20	47	51
17	22	47	53
17	23	47	55
**17**	**24**	47	57
17	25	47	59
17	26	**47**	**60**
17	27	47	67

Deletion starts at exon	Deletion ends at exon	Deletion starts at exon	Deletion ends at exon
17	28	47	69
17	29	47	75
17	30	47	78
17	31	48	48
17	32	48	49
17	33	48	51
17	34	48	53
17	35	48	55
17	36	48	57
17	37	48	59
17	38	48	60
17	39	48	67
17	40	48	69
17	41	48	75
17	42	48	78
17	44	49	49
17	46	49	51
17	47	49	53
**17**	**48**	49	55
17	49	49	57
17	51	49	59
17	53	49	60
17	55	49	67
17	57	49	69
17	59	49	75
17	60	49	78
17	67	50	51
17	69	**50**	**53**
17	75	50	55
17	78	50	57
18	43	50	59
18	45	50	60
18	54	50	67
18	56	50	69
18	62	50	75
18	65	50	78
18	68	**51**	**52**
18	70	51	58
18	71	51	61
18	72	51	63
18	73	51	64
18	74	51	66

Deletion starts at exon	Deletion ends at exon	Deletion starts at exon	Deletion ends at exon
**19**	**20**	51	76
19	22	51	77
19	23	52	53
19	24	52	55
19	25	52	57
19	26	52	59
19	27	52	60
19	28	52	67
19	29	52	69
19	30	52	75
19	31	52	78
19	32	53	58
19	33	53	61
19	34	53	63
19	35	53	64
19	36	53	66
19	37	53	76
19	38	53	77
19	39	54	55
19	40	54	57
19	41	54	59
19	42	54	60
19	44	54	67
19	46	54	69
19	47	54	75
19	48	54	78
19	49	55	56
19	51	55	62
19	53	55	65
19	55	55	68
19	57	55	70
19	59	55	71
19	60	55	72
19	67	55	73
19	69	55	74
19	75	56	57
19	78	56	59
20	21	56	60
**20**	**50**	56	67
20	52	56	69
20	58	56	75
20	61	56	78

Deletion starts at exon	Deletion ends at exon	Deletion starts at exon	Deletion ends at exon
20	63	57	62
20	64	57	65
20	66	57	68
20	76	57	70
20	77	57	71
21	22	57	72
21	23	57	73
21	24	**57**	**74**
21	25	**58**	**59**
21	26	58	60
21	27	58	67
21	28	58	69
21	29	58	75
21	30	58	78
21	31	59	61
21	32	59	63
21	33	59	64
21	34	59	66
21	35	59	76
21	36	59	77
21	37	**60**	**60**
21	38	60	67
21	39	60	69
21	40	60	75
21	41	60	78
21	42	61	67
21	44	**61**	**69**
21	46	61	75
21	47	61	78
21	48	62	63
21	49	62	64
21	51	62	66
21	53	62	76
21	55	62	77
21	57	63	65
21	59	63	68
21	60	63	70
21	67	63	71
21	69	63	72
21	75	63	73
21	78	63	74
22	50	64	64

Deletion starts at exon	Deletion ends at exon	Deletion starts at exon	Deletion ends at exon
22	52	64	66
22	58	64	76
22	61	64	77
22	63	65	66
22	64	65	76
22	66	65	77
22	76	66	68
22	77	66	70
23	23	66	71
23	24	66	72
23	25	66	73
23	26	66	74
23	27	67	76
23	28	67	77
23	29	68	69
23	30	68	75
23	31	68	78
23	32	69	70
23	33	69	71
23	34	69	72
23	35	69	73
23	36	69	74
23	37	70	75
23	38	70	78
23	39	71	71
23	40	71	72
23	41	71	73
23	42	71	74
23	44	72	72
23	46	72	73
23	47	72	74
23	48	73	73
23	49	73	74
23	51	74	74
23	53	76	78
23	55	77	77
